# Role of RIPK1 in SMAC mimetics-induced apoptosis in primary human HIV-infected macrophages

**DOI:** 10.1038/s41598-021-02146-w

**Published:** 2021-11-25

**Authors:** Ramon Edwin Caballero, Simon Xin Min Dong, Niranjala Gajanayaka, Hamza Ali, Edana Cassol, William D. Cameron, Robert Korneluk, Michel J. Tremblay, Jonathan B. Angel, Ashok Kumar

**Affiliations:** 1 Department of Biochemistry, Microbiology, and Immunology, Faculty of Medicine, University of Ottawa, Ottawa, ON Canada; 2 Division of Virology, Apoptosis Research Centre, Children’s Hospital of Eastern Ontario Research Institute, 401 Smyth Road, Research Building 2, University of Ottawa, Ottawa, ON K1H 8L1 Canada; 3 Department of Health Sciences, Carleton University, Ottawa, ON Canada; 4 Division of Infectious Diseases, The Ottawa Hospital Research Institute, Ottawa, ON Canada; 5 Centre de recherche du CHU de Québec-Université Laval, Université Laval, Québec City, QC Canada; 6 Department of Pathology and Laboratory Medicine, Faculty of Medicine, University of Ottawa, Ottawa, ON Canada

**Keywords:** Immunology, Microbiology, Diseases, Medical research, Molecular medicine, Pathogenesis

## Abstract

Macrophages serve as viral reservoirs due to their resistance to apoptosis and HIV-cytopathic effects. We have previously shown that inhibitor of apoptosis proteins (IAPs) confer resistance to HIV-Vpr-induced apoptosis in normal macrophages. Herein, we show that second mitochondrial activator of caspases (SMAC) mimetics (SM) induce apoptosis of monocyte-derived macrophages (MDMs) infected in vitro with a R5-tropic laboratory strain expressing heat stable antigen, chronically infected U1 cells, and ex-vivo derived MDMs from HIV-infected individuals. To understand the mechanism governing SM-induced cell death, we show that SM-induced cell death of primary HIV-infected macrophages was independent of the acquisition of M1 phenotype following HIV infection of macrophages. Instead, SM-induced cell death was found to be mediated by IAPs as downregulation of IAPs by siRNAs induced cell death of HIV-infected macrophages. Moreover, HIV infection caused receptor interacting protein kinase-1 (RIPK1) degradation which in concert with IAP1/2 downregulation following SM treatment may result in apoptosis of macrophages. Altogether, our results show that SM selectively induce apoptosis in primary human macrophages infected in vitro with HIV possibly through RIPK1. Moreover, modulation of the IAP pathways may be a potential strategy for selective killing of HIV-infected macrophages in vivo.

## Introduction

Macrophages are permissive to productive infection with HIV and a source of viral progeny for transmission to other cell types such as T cells^1–4^. In contrast to the typical depletion of CD4 + T cells, macrophages do not decline in number, are resistant to apoptosis, survive viral replication, and harbor unintegrated and integrated viral DNA in a state of latency^1,5–9^. In patients on antiretroviral therapy (ART), macrophages serve as reservoirs as HIV persists in these cell and is shielded against various host anti-viral responses; while subsequently responding poorly to ART ^1,2,5,9–13^. Since HIV-infected macrophages are not cleared by CD8^+^ T cells, neither current ART nor the immune system is able to effectively eliminate this reservoir^14^.

While several recent studies support that macrophages serve as a major non-T cell HIV reservoir^15–18^, the role of macrophages in HIV infection and persistence has been conclusively demonstrated by employing humanized bone marrow, liver, thymus (BLT) and myeloid only mice (MoM) (mice containing myeloid cells devoid of T cells)^2,19^. Therefore, to completely eradicate HIV in individuals on ART, it is imperative to eliminate both CD4 + T cells and myeloid tissue reservoirs. Most research to date has focused on eliminating the latent reservoir of CD4 + T cells by employing strategies to reactivate HIV in T cells and elimination of reactivated HIV-infected cells by host immunity^20,21^. However, approaches towards killing HIV-infected macrophages in vitro or in vivo are not well studied. Recently, a few attempts have been made to clear macrophage reservoirs by targeting infected macrophages with antagonists for CSF-1 receptor^22^ and cellular inhibitors for apoptosis (cIAP) proteins^23^, galactin-3^24^, TREM-1^25^ and oncolytic viruses^26^.

To devise strategies to eliminate HIV-infected macrophages, it is imperative to identify apoptosis-related genes and signaling proteins involved in resistance of HIV-infected macrophages to apoptosis. The mechanism underlying this resistance may be related to the differential expression of pro- and anti-apoptotic genes including cIAP proteins. The role of IAPs has been studied by employing their synthetic antagonists, the second mitochondria-derived activator of caspases (SMAC) mimetics (SMs). SMs are small peptides that mimic the N-terminal four amino-acid sequence of SMAC and bind to XIAP and cIAP1/2 leading to caspase activation or enhanced E3 ligase activity to promote auto-ubiquitination and proteasomal degradation eventually leading to the repression of anti-apoptotic functions of IAP proteins and cell death^27–29^. Recently, cIAP1/2 and survivin, another members of the IAP family, were suggested to be involved in survival of HIV-infected CD4 + T cells^30,31^ and macrophages from HIV-infected individuals^23^. SMAC mimetics (LCL161, and birinapant) were shown to kill HIV-infected macrophages without increasing bystander cell death via autophagy-dependent apoptosis^23^. In addition, IAPs have been implicated in the reversal of HIV latency in CD4 + T cells^32–34^. Using HIV-Vpr as an apoptosis-inducing agent, we have shown a protective role for IAP genes in resistance to cell death in macrophages^35–37^. Therefore, strategies based on suppressing IAPs by employing SMs, may be useful in killing HIV-infected macrophages. Herein, we show that SMs induced apoptosis in in vitro HIV-infected macrophages and that this killing may occur through the concomitant down regulation of both IAPs and receptor interacting protein kinase-1 (RIPK1).

## Results

### SMs induce cell death in HIV-infected myeloid U1 cells but not in counterpart uninfected U937 cells

**Figure 1:**
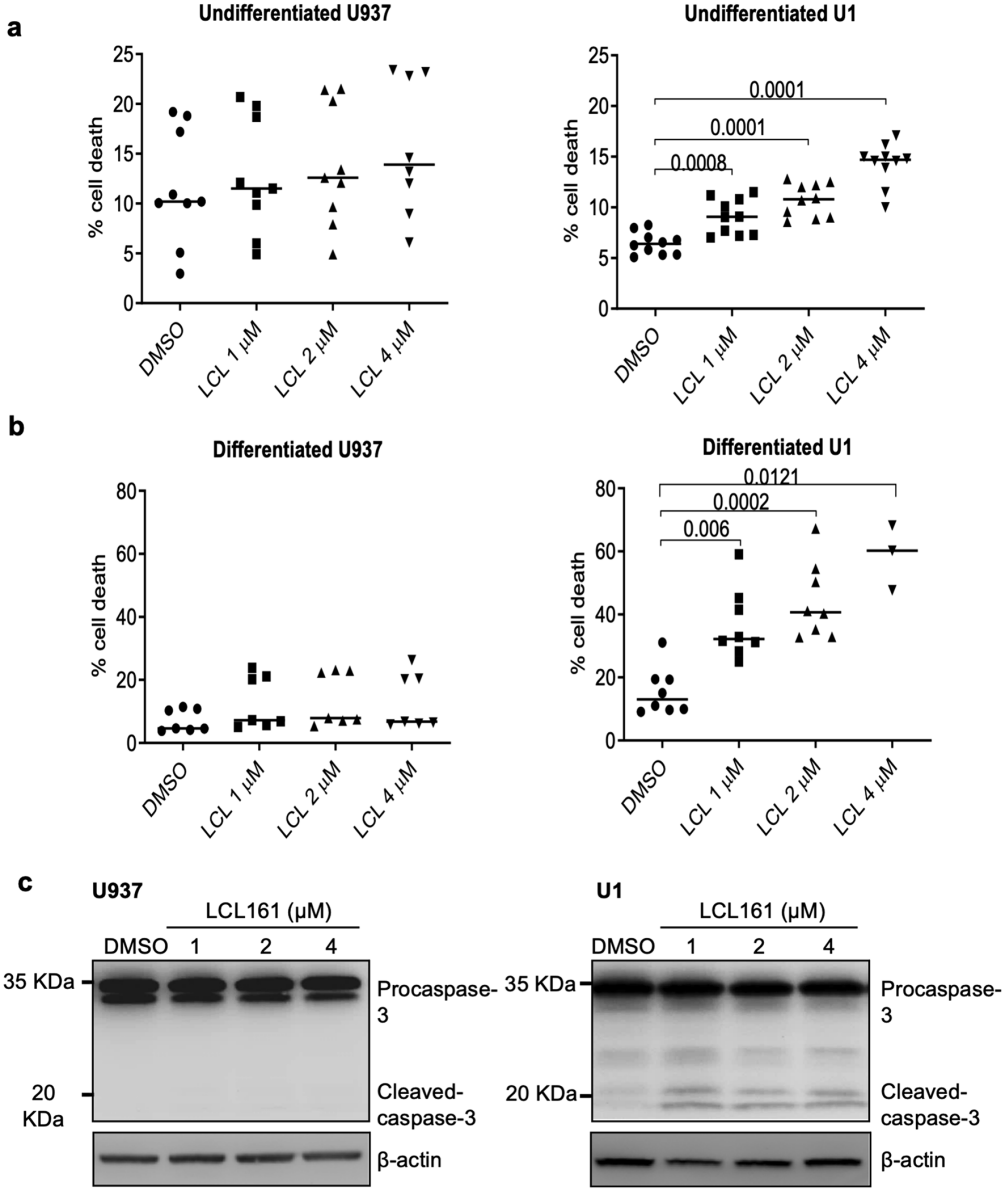
SM induces cell death of HIV-infected myeloid cells. (**a**) U937 (technical replicate (tn) = 9) and chronically infected counterpart U1 cells (5.0 × 10^5^) (tn = 10) were treated with LCL161 at 1, 2, and 4 μM for 48 h. (**b**) PMA differentiated U937 (tn = 7) and U1 cells (tn = 11) were treated with SM LCL161 at 1, 2, and 4 μM for 48 h. Cell death was assessed by intracellular PI staining. The *p*-values were calculated using two-tailed Mann–Whitney U test. (**c**) U937 (tn = 2) and U1 (tn = 4) were treated with increasing concentration of LCL161 for 48 h and cytosolic fractions were collected and 30 µg of total proteins were subjected to western immunoblotting. The membranes were probed with antibodies specific for caspase-3. The uncropped western blot images are in Supplementary Fig. 6. The densitometric readings for figure (**c**) are shown in Supplementary Fig. 11A.

We have previously shown that the cIAP1/2 play a protective role in mediating survival of macrophages in response to Vpr-induced cell death^35–37^. Here, we hypothesized that SMs, the IAPs antagonists, will induce apoptosis in HIV-infected macrophages. U1 cells are chronically infected with two copies of proviral HIV DNA and show minimal constitutive expression of the virus. PMA stimulation of U1 cells induces HIV production and terminal differentiation along the mononuclear phagocytic lineage^38^ similar to HIV infection of primary macrophages^39^. Thus, we first evaluated the effects of SMs on apoptosis in undifferentiated and PMA-differentiated U937 and U1 cells. U1 cells and uninfected counterpart U937 cells were treated with SM-LCL161 (LCL161) followed by assessment of cell death by PI staining and flow cytometry. LCL161 treatment induced significant cell death in undifferentiated U1 cells (*p*-value = 0.008, DMSO *vs* LCL 1 μM) but not in U937 cells (Fig. 1a). Like the effect of SM on undifferentiated U1 cells, LCL161 induced significant cell death in PMA-differentiated primary macrophage-like U1 cells (*p*-value = 0.006, DMSO *vs* LCL 1 μM) but not in PMA-differentiated U937 cells (Fig. 1b). Apoptosis of HIV-infected U1 cells was further confirmed by showing cleavage of caspase-3 in U1 but not in U937 cells by immunoblotting (Fig. 1c).

### SMs induce cell death in in vitro HIV-infected MDMs and MDMs derived from HIV-infected patients

**Figure 2:**
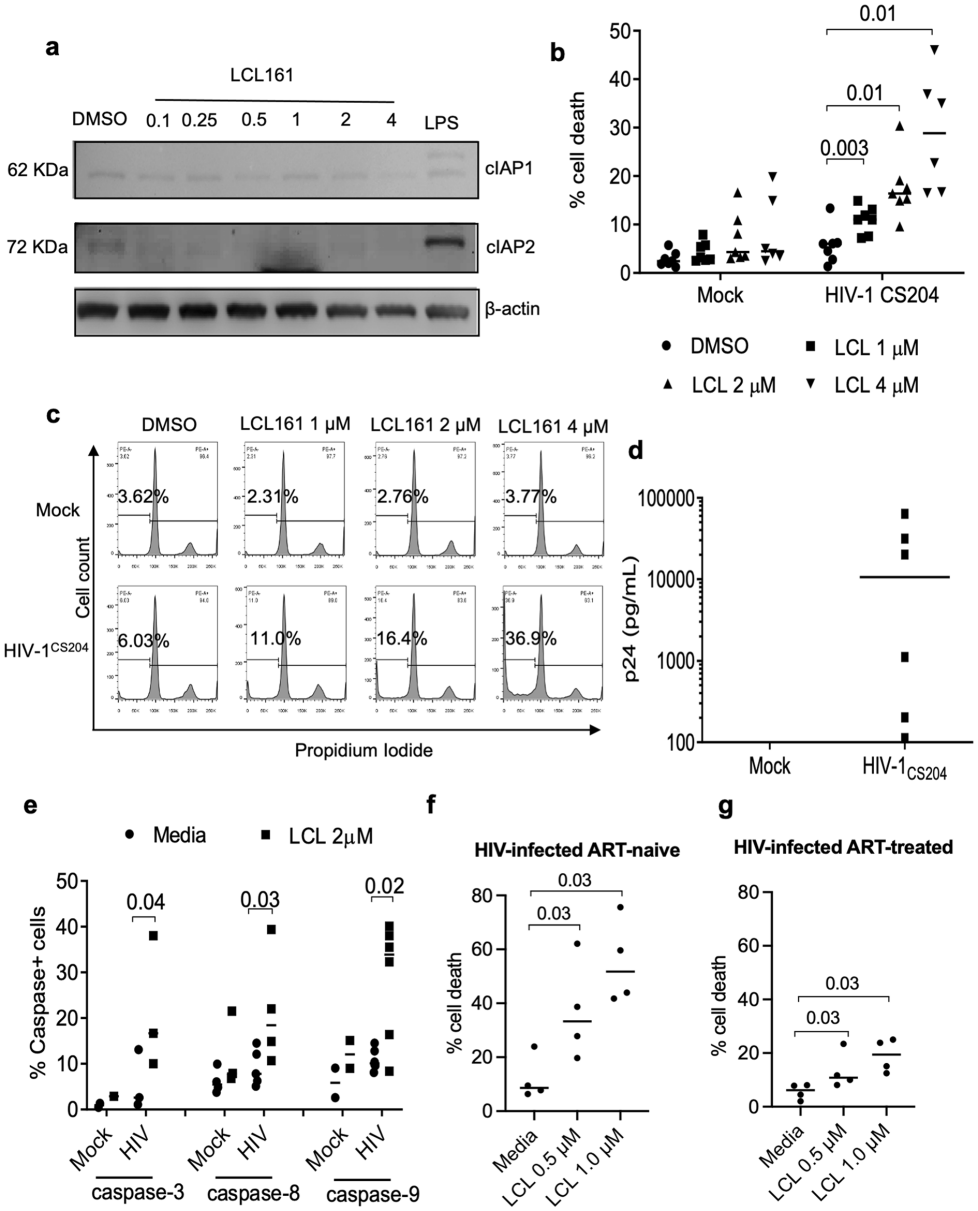
SM induces cell death of HIV-infected MDMs (**a**). MDMs were treated with increasing concentration of LCL161 for 48 h and cytosolic fractions were collected. 30 µg of total proteins were subjected to western immunoblotting to probe for cIAP1 and cIAP2. (**b**) Human MDMs were in vitro infected with HIV-1 CS204 (100 ng p24/well) for 7 days (n = 4). The cells were then treated with LCL161 for 48 h and cell death was assessed by PI staining and flow cytometry. The representative histograms are shown in (**c**). (**d**) After 7 days of infection, supernatants were analyzed for p24 by ELISA (n = 6). (**e**) Mock and HIV-infected MDMs treated with SM for 48 h and caspase-3 (n = 3), -8 (n = 4), and -9 (n = 6) positive cells were analyzed by flow cytometry. MDMs generated from naïve (**f**) and ART-treated (**g**) HIV-individuals were treated with LCL161 for 48 h. Cell death was assessed by PI staining and flow cytometry. The *p*-values were calculated using two-tailed Mann–Whitney U test. The uncropped western blot images are in Supplementary Fig. 7. The densitometric readings for figure (**a**) are shown in Supplementary Fig. 11B.

To validate the above findings in primary MDMs, we first verified the functional activity of SM by treating HIV-infected MDMs with LCL161 and observed degradation of both cIAP1 and cIAP2 (Fig. 2a) as previously reported^36,40^. LCL161 induced significant cell death of in vitro HIV-1 CS204-infected (*p*-value = 0.003, DMS0 *vs* LCL 1 μM) MDMs but not in mock-infected MDMs (Fig. 2b). Representative histograms of the intracellular PI staining are shown (Fig. 2c). The p24 values in MDMs infected with HIV-1 CS204 for 7 d are shown in Fig. 2d. SM-induced cell death experiments were conducted at 48 h post-treatment with LCL161 as higher % of cell death was observed at 48 h compared to 24 h post treatment (Supp Fig. 1a). To determine whether SM-induced cell death in in vitro HIV-infected MDM was due to apoptosis, caspase activation was quantified based on the fluorescent signal of cleaved caspase substrates. Treatment of HIV-1 CS204-infected MDM with LCL161 showed activation of caspases 3, 8, and 9 in contrast to the mock-infected MDM (Fig. 2e). A representative histogram for the induction of caspase 3, 8 and 9 is shown (Supp Fig. 2).

Further, to determine whether macrophages derived from HIV-infected patients are similarly prone to SM-induced cell death, MDMs were generated from ART-treated and ART-naïve HIV-infected patients and treated with LCL161. Consistent with in vitro infection studies, ex vivo derived MDMs from treatment-naïve (*p*-value = 0.03, DMS0 *vs* LCL 1 μM) and ART-treated patients (*p*-value = 0.03, DMS0 *vs* LCL 1 μM) showed a significantly increased susceptibility to LCL161-induced cell death in a dose-dependent manner (Fig. 2f, g). Moreover, ART treatment did not affect the expression of cIAP1 and cIAP2 in uninfected or HIV-infected macrophages (Supp Fig. 1b) suggesting that ART treatment did not affect the IAP pathway in uninfected or HIV-infected macrophages.

### SMs specifically kill HIV-infected MDMs

**Figure 3:**
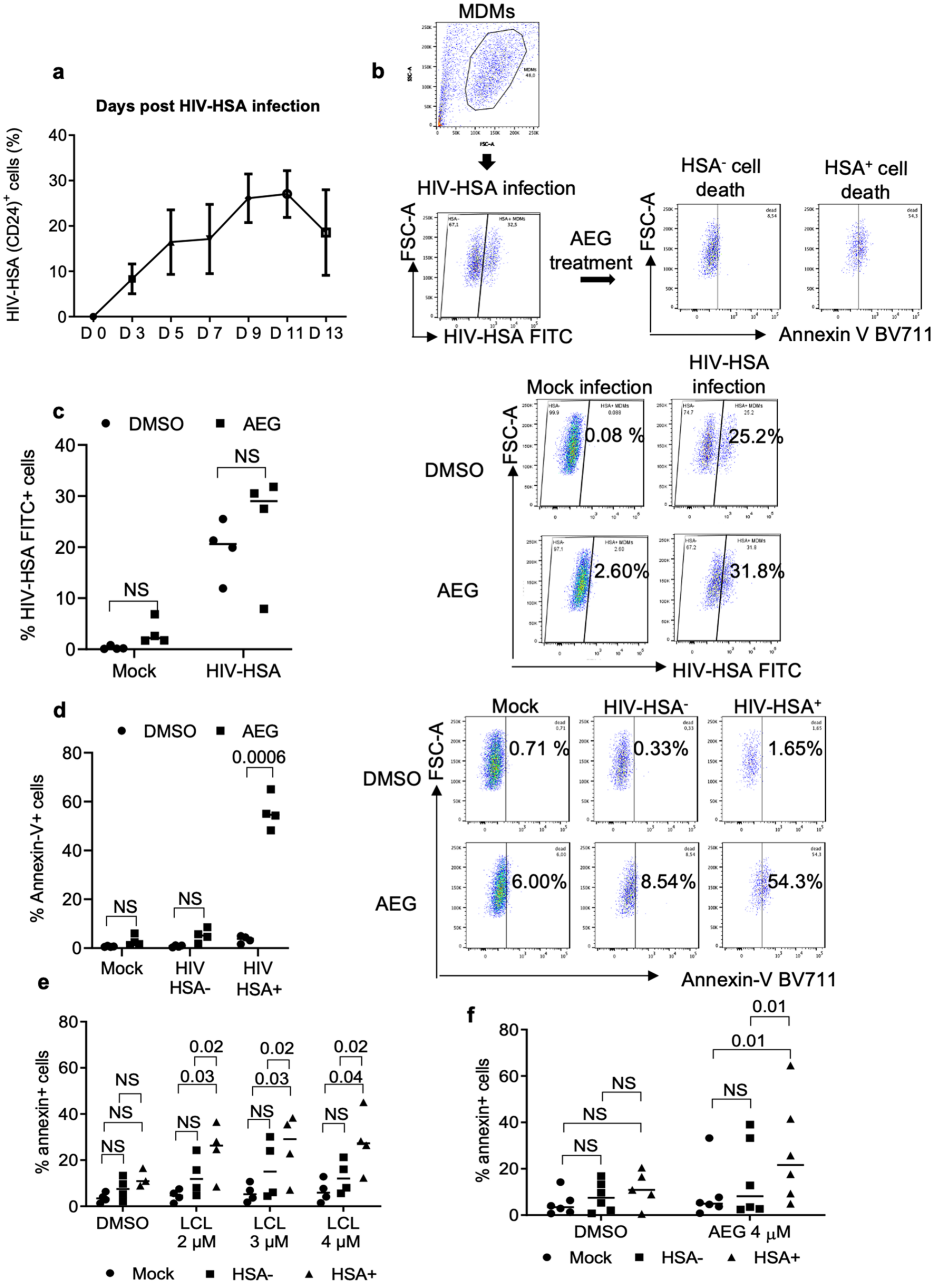
SM specifically induces cell death of HIV-HSA-infected MDMs. (**a**). MDMs from healthy donors were in vitro infected with HIV-HSA (100 ng p24/well). Infection rate over time was detected using anti-CD24 (HSA)-FITC antibody by flow cytometry. (**b**) Gating strategy for the detection of apoptosis by Annexin-V staining of HIV-infected total (HSA + and HSA −) MDMs. (**c**) The in vitro HIV-HSA-infected MDMs 7 days post-infection were treated with 5 µM of AEG40730 (AEG) for 72 h and degree of infection was determined by anti-CD24 (HSA) antibody staining and flow cytometry (left panel) (n = 4). Representative scatter plots of HIV-HSA FITC + cells are shown (right panel). (**d**) Degree of cell death after AEG treatment of mock, HSA + and HSA-HIV-infected cells was assessed by annexin-V-BV711 staining and flow cytometry (left panel) (n = 4). Representative scatter plots show annexin-V-BV711 cells (right panel). LCL161 (**e**) and AEG 40730 (**f**) specifically kill HIV-HSA-infected cells but not HIV-HSA negative MDM. MDMs were infected with HIV-HSA for 11 days followed by treatment with either LCL161 or AEG40730 for another 48 h followed by analysis of cell death by PI staining (n = 5). The *p* values were calculated using two tailed Mann–Whitney U test.

The frequency of HIV-infected macrophages in in vitro HIV-infected macrophages may not exceed 10%. However, the HIV-infected myeloid cells are rarely detected in the blood of HIV-infected individuals^41^; hence the frequency of HIV-infected macrophages in ex vivo derived macrophages from the HIV-infected patients may be extremely low. But the above results show killing of 20–30% macrophages (Fig. 2b, f, g) following treatment with SMs. Therefore, it was important to ascertain whether SMs were killing HIV-infected cells alone, uninfected bystander cells alone or both. To examine this, we employed a R5 laboratory strain of HIV-1, HIV-Bal-HSA (HIV-HSA), expressing mouse HSA (CD24). Expression of HSA by HIV-infected cells can be used to quantify infected cells by flow cytometry using FITC-conjugated anti-mouse HSA antibody^42^. HIV-HSA infection rates in MDMs ranged from 5 to 30% over 15 d post infection depending upon the donor variability (Fig. 3a). Flow cytometric analysis of HIV-HSA-infected macrophages revealed two distinct HSA-positive (HSA +) and HSA-negative (HSA −) populations of macrophages (Fig. 3b). Following treatment with SM-AEG40730 (AEG40730), HIV-HSA-infected and HIV-HSA negative macrophages (uninfected, HIV exposed bystander macrophages) were analyzed for infection rate (% HSA + cells) and apoptosis as quantified by counter staining with Annexin-V labelled with BV711 expression. The gating strategy is shown in Fig. 3b. Analysis of infection rate (% HIV-HSA FITC + cells) following AEG40730 treatment did not reveal significant differences between control DMSO and AEG40730 treated macrophages (Fig. 3c, left panel). As a control, AEG40730 treatment did not show significant increase in non-specific FITC-HSA autoflourescence in mock uninfected macrophages (Fig. 3c, left panel). Representative histograms are shown (Fig. 3c, right panel). Quantification of Annexin-V positive, HSA + and HSA − MDMs revealed that AEG40730 killed a significantly higher number of HIV-HSA-infected macrophages compared to the control DMSO-treated HSA-infected macrophages (*p*-value = 0.0006, DMS0 *vs* AEG) (Fig. 3d, left panel). In contrast, there was no significant difference in cell death between DMSO-treated and AEG40730-treated mock uninfected and HIV-HSA-negative uninfected macrophages (Fig. 3d, left panel). Representative histograms showing killing of HSA + and HSA − macrophages is shown (Fig. 3d, right panel).

### SMs kill significantly higher numbers of HIV-infected macrophages than uninfected bystander cells

The HIV-exposed but uninfected bystander cells play a critical role in HIV pathogenesis and disease progression by causing selective depletion of CD4 + T cells, leading to immunodeficiency^43^. Although HIV infection of T cells and macrophages differ with respect to cell death in vivo and in vitro^1,14,17,18^, uninfected bystander macrophages may also play a crucial role in HIV pathogenesis. Therefore, we determined if SMs induce apoptosis of HIV-exposed uninfected bystander macrophages. AEG40730 and LCL161 killed significantly high numbers of HIV-HSA-expressing (HIV-infected) cells compared to either the mock or HSA-negative (HIV-uninfected/bystander, HIV-exposed) cells (Fig. 3e, f) (*p*-value = 0.04, LCL 4 μM, Mock *vs* HSA + ; *p*-value = 0.02 LCL 4 μM, HSA − *vs* HSA + ; *p*-value = 0.01, AEG 4 μM, Mock *vs* HSA + ; *p*-value = 0.01 AEG 4 μM, HSA − *vs* HSA +). While killing of HSA-negative (HIV-uninfected/bystander) cells was somewhat higher than levels seen in mock-infected cells, these differences were not significant suggesting that SMs specifically kill HIV-infected macrophages (Fig. 3e, f).

### Knocking down IAP genes results in specific killing of HIV-infected MDM

**Figure 4:**
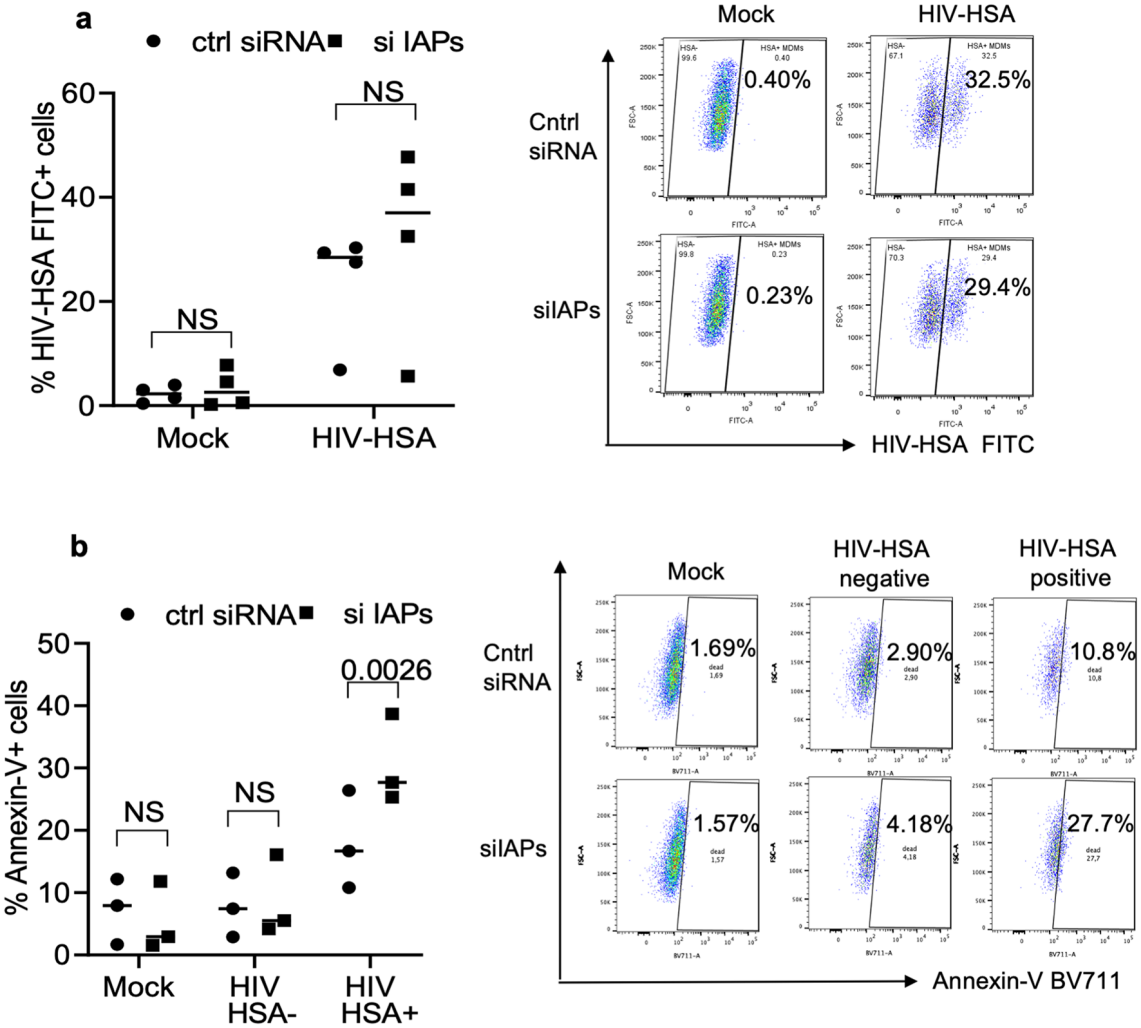
IAP siRNAs specifically induce cell death of HIV-HSA-infected MDMs. MDMs were in vitro infected with HIV-HSA for 7 days. The cells were transfected with non-targeting control siRNA or IAPs siRNAs. (**a**) After 72 h of transfection, degree of infection was assessed by anti-CD24 (HSA) antibody staining (left panel). Representative scatter plots show HIV-HSA FITC + cells (right panels). (**b**) After 72 h of transfection, cell death of mock, HIV-HSA− and HIV-HSA+ cells were detected by Annexin-V-BV711 and flow cytometry (left panel) (n = 4). Representative scatter plots show annexin-V-BV711 + cells (right panel). The *p*-values were calculated using two-tailed Mann Whitney U test.

To confirm the involvement of IAPs, we employed cIAP1/2 siRNAs as previously^35–37^. Flow cytometry of HIV-HSA-infected macrophages from healthy donors revealed two distinct HSA-positive (HSA +) and HSA-negative (HSA −) populations (Fig. 4a, right panel). Following transfection with siRNAs for 72 h, HIV-HSA-infected and HIV-HSA negative uninfected macrophages (HIV exposed bystander macrophages) were quantified for infection rate (% HSA + cells) and apoptosis using Annexin-V labelled with BV711 antibodies as above. The gating strategy applied was same as shown in Fig. 3b. Analysis of infection rate (% HIV-HSA FITC + cells) following transfection with control non-targeting siRNA or cIAP1/2 siRNAs did not show significant differences in mock and HIV-HSA-infected macrophages (Fig. 4a). Furthermore, control or cIAP1/2 siRNAs did not show any increase in non-specific FITC-HSA autoflourescence in mock uninfected macrophages (Fig. 4a). Representative histograms are shown (Fig. 4a, right panel).

Quantification of Annexin-V positive HSA + and HSA − MDMs revealed that knocking down cIAP1/2 by siRNAs killed significantly higher numbers of HIV-HSA+ macrophages compared to the HIV-HSA-infected cells treated with non-targeting siRNAs (Fig. 4b) (*p*-value = 0.0026, HIV-HSA+ , ctrl siRNA *vs* si IAPs). In contrast, there was no significant difference in cell death between control and IAP siRNA transfected mock uninfected and HIV-HSA-negative uninfected macrophages (Fig. 4b). Representative histograms are shown in Fig. 4b, right panel. The functional activity of the same cIAP1/2 siRNA has been shown by us previously^35–37^.

### SM do not affect HIV replication in HIV-infected macrophages

**Figure 5:**
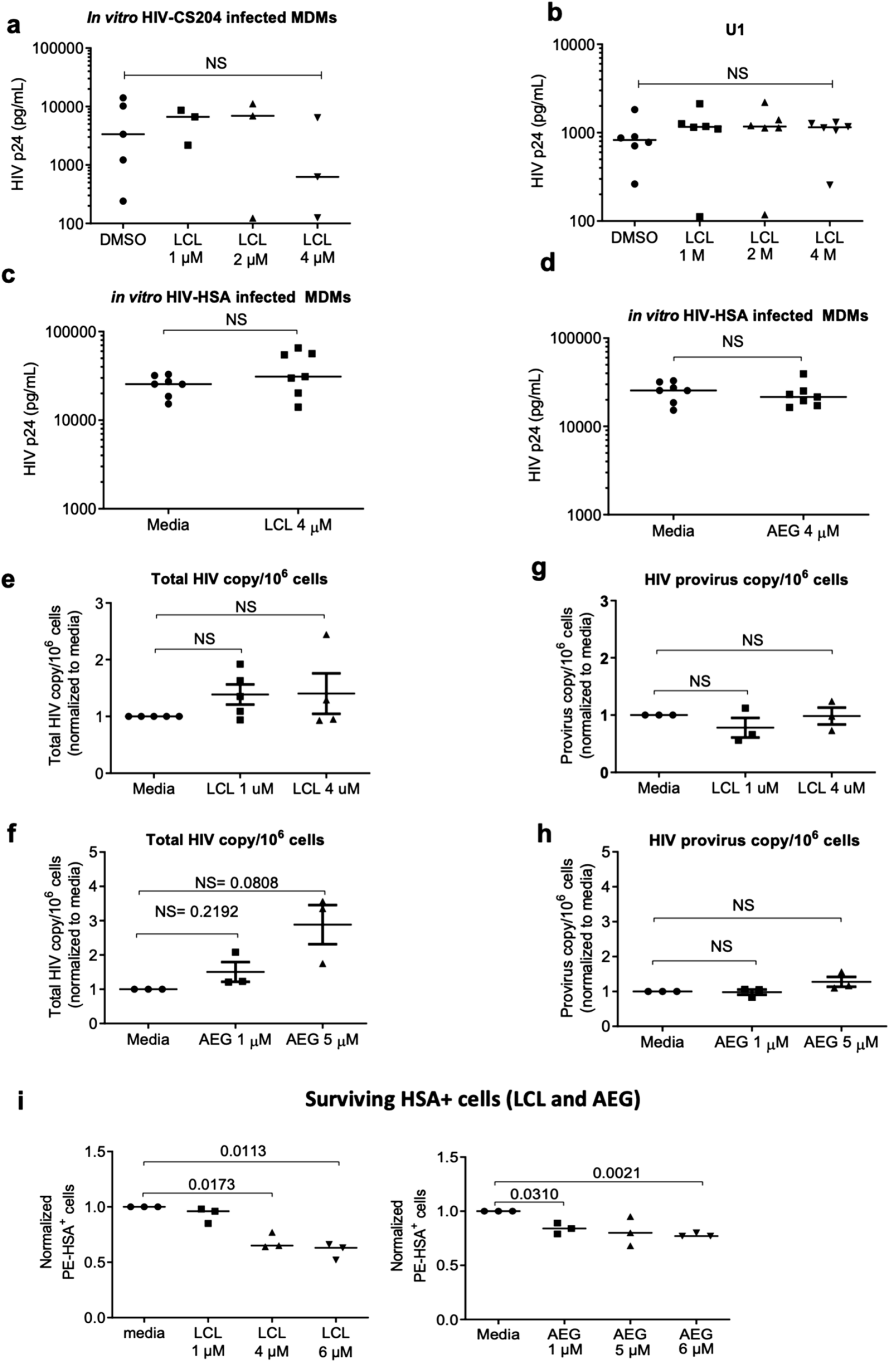
SMs do not affect HIV replication in HIV-infected macrophages. (**a**) In vitro HIV-1 CS204 infected MDMs (n = 5) and (**b**) undifferentiated U1 (tn = 6) cells were treated with increasing dose of LCL161. After 48 h, supernatants were quantified for p24 by ELISA. In vitro HIV-HSA infected MDMs were treated with LCL161 (n = 7) (**c**) and AEG40730 (n = 7) (**d**) for 4 d and p24 in the supernatants was analyzed by ELISA. MDMs were infected in vitro with HIV-1 CS204 for 7 d were treated with LCL161 and AEG40730. Whole cell lysates were analyzed for Total HIV-DNA (**e** and **f**, n = 5) and integrated proviral HIV-DNA (**g** and **h**, n = 3) using ultra sensitive qRT-PCR. (**i**) HIV-HSA infected MDMs were treated with LCL161 and AEG40730 for 7 days. Dead cells were thoroughly removed from the culture well by washing with DPBS. Surviving adherent cells were collected and were analyzed for HIV-HSA infection by anti-HSA staining and flow cytometry (n = 3). The *p*-values were calculated using two-tailed Mann–Whitney U test. (NS: no significance).

Apoptosis has been shown to induce viral activation and replication in latently infected U1 and ACH2 cell lines^44^. In addition, Pache et al. have shown that SMs enhanced viral transcription in infected HEK293T cells and CD4^+^ T cells via NF-κB dependent signaling^32^**.** To determine if SMs affect HIV replication in macrophages, in vitro HIV CS204-infected MDM were treated with SM-LCL161 for 48 h followed by analysis of p24 secretion. LCL161 treatment at different concentrations did not significantly affect p24 levels in HIV-infected MDM (Fig. 5a). Similar results were seen with LCL161-treated U1 cells (Fig. 5b) and LCL161 or AEG40730 treated HIV-HSA infected macrophages (Fig. 5c, d). This was confirmed by analyzing the expression of total HIV-DNA and integrated HIV-DNA following LCL161 and AEG40730 treatment of MDMs infected with HIV-1 CS204. LCL161 and AEG40730-treated macrophages did not show an increase in total HIV DNA levels (Fig. 5e, f) or levels of integrated (provirus) HIV-DNA (Fig. 5g, h) at any concentration, suggesting that LCL161 and AEG40730 treatment did not affect HIV replication following HIV infection. We did not observe a drop in the percentage of infected cells and p24 levels following exposure to SMs as Annexin-V detects early stage of apoptosis and the cells are still alive and intact and secreting p24. Annexin positive cells we observed will eventually die at a later stage. Consistent with this, when we analyzed cell death after 7 days of exposure to SMs, we did observe a significant drop in the percentage of infected cells following LCL161 (*p*-value = 0.0173, Media *vs* LCL 4 μM) and AEG40730 (*p*-value = 0.0021, Media *vs* AEG 5 μM) treatment of HIV-infected macrophages (Fig. 5i).

### TNF-α mediates SM-induced apoptosis in U1 cells

**Figure 6:**
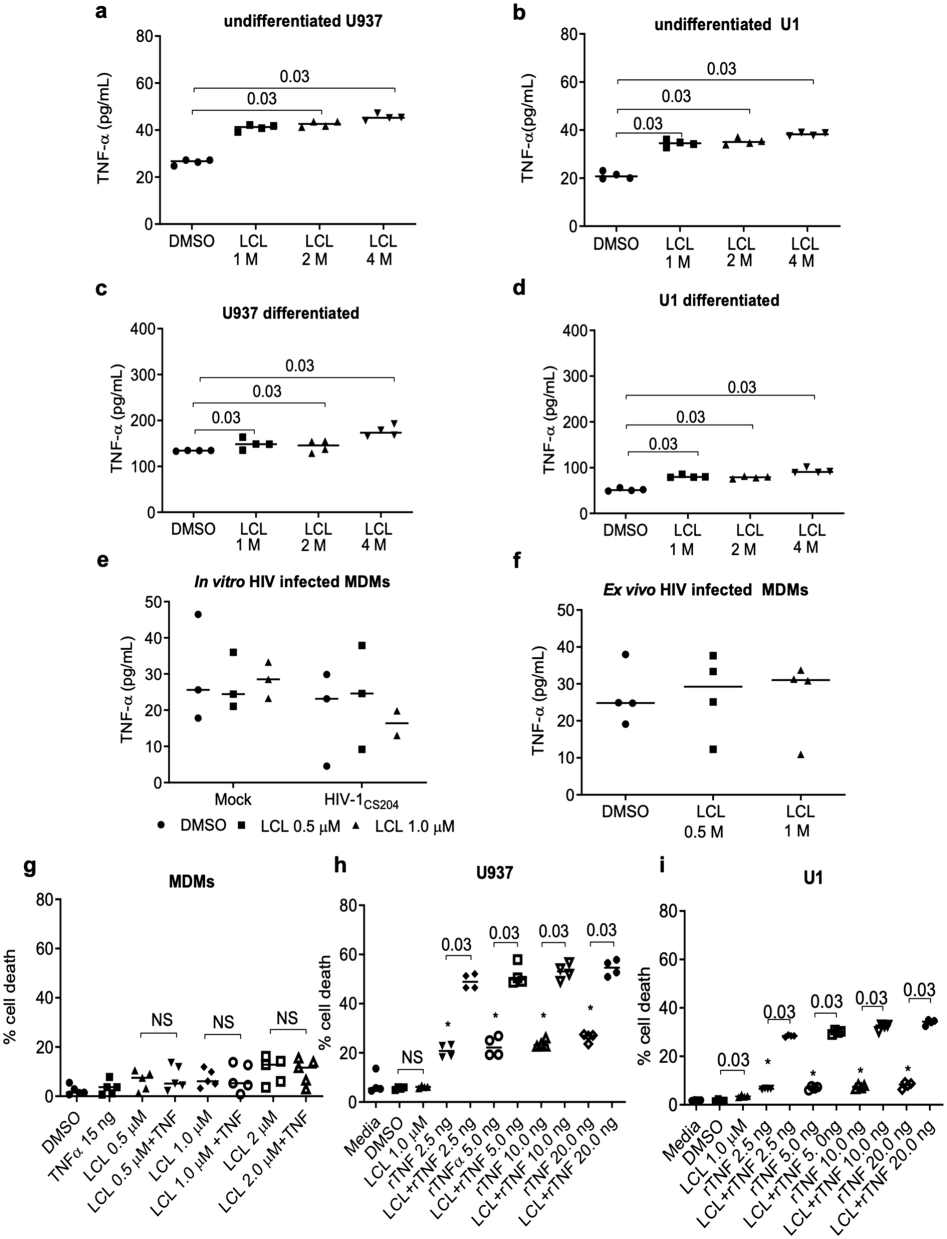
TNF mediates SM-induced apoptosis in U1 cells but not in HIV-infected MDM. SMs induce TNF secretion in undifferentiated and differentiated U937 and U1 cells. (**a**) Undifferentiated U937 (tn = 4) and (**b**) U1 (tn = 4), (**c**) differentiated U937 (tn = 4), and (**d**) U1 (tn = 4) cells were treated with LCL161 for 48 h. The supernatants were analyzed for TNF production by ELISA. SM treatment of HIV-infected MDMs does not induce TNF production. (**e**). Human MDMs were in vitro infected with HIV-1 CS204 (100 ng p24 / well) for 7 days followed by the addition of LCL161 for 48 h (n = 3). (**f**) MDMs ex vivo derived from HIV patients were treated with LCL161 for 48 h (n = 4). Supernatants were analyzed for TNF production by ELISA. The positive control value for TNF production following LPS stimulation of M1 macrophages was 47,140 pg/ml. (**g**) MDMs were treated with LCL161 for 2 h followed by the addition of rTNF for 48 h. Intracellular PI staining and flow cytometry were used to assessed levels of cell death (n = 5). (**h**) U 937 (tn = 4) and (**i**) U1 (tn = 4) were treated with either LCL161 alone, rTNF alone or with LCL161 and various concentrations of rTNF for 48 h followed by analysis of cell death by PI staining and flow cytometry. The *p*-values were calculated using two tailed Mann–Whitney U test.

SM-induced cell death of various tumor cells is mediated by endogenously produced TNF-α (TNF) following SM treatment through the activation of the non-canonical NF-κB pathway^45,46^. To determine if SM-induced apoptosis in HIV-infected MDM is due to endogenous TNF production, LCL161-treated U937, U1 cells and in vitro HIV-infected MDM were analyzed for TNF secretion. LCL161 treatment resulted in low level although significant TNF production (*p*-value = 0.03, DMSO *vs* LCL 1–4 μM) in undifferentiated and differentiated U937 and U1 cells compared to DMSO-treated control cells (Fig. 6a–d). In contrast, LCL161 treatment did not induce significant TNF production in both in vitro mock- and HIV-infected MDM (Fig. 6e). Similar to in vitro HIV-infected macrophages from healthy donors, ex vivo derived MDMs from HIV-infected patients did not produce significantly higher levels of TNF following LCL161 treatment compared to the untreated negative controls (Fig. 6f).

To evaluate the impact of TNF in SM-induced apoptosis of MDM, LCL161-treated MDM were stimulated with recombinant TNF (rTNF) followed by analysis of cell death using PI staining. Treatment of MDMs with LCL161 alone or with rTNF did not result in significant cell death (Fig. 6g). In contrast, rTNF either alone or in combination with LCL161 induced significant cell death in U937 and U1 cells (Fig. 6h, i), similar to that observed in various tumor cells^47,48^. These results suggest that SM-mediated killing of HIV-infected primary macrophages may be independent of endogenously produced TNF. However, the involvement of TNF in SM-induced cell death can be confirmed only following silencing of TNFR1 & TNFR2 in HIV-infected macrophages which may not be technically feasible at present.

### HIV-infected MDM do not develop M1 phenotype before or after SMAC mimetics treatment

We have shown that SMs selectively kill IFN-γ-generated M1 macrophages^49^ (Supp Fig. 3). Hence, SM-induced cell death of HIV-infected MDMs may be due to the acquisition of M1 phenotype following HIV infection. To determine whether HIV-infected MDMs develop a M1 phenotype before or after SM treatment, cytokine array analysis for the following cytokines was performed: IL-17F, GM-CSF, IFN-γ, IL-10, CCL20/MIP3a, IL-12p70, IL-13, IL-15, IL-17a, IL-22, IL-9, IL-1β, IL-33, IL-21, IL-23, IL-5, IL-6, IL-17ε/IL-25, IL-27, IL-31, TNFα, TNFβ, and IL-28A. HIV-infected MDM secreted significantly high levels of CCL20/MIP3-α (*p*-value = 0.04), IL-6 (*p*-value = 0.04), and TNFα (*p*-value = 0.04) compared to the mock control. But there was no difference in the secretion of IL-10, IL-21, IL-13, and IL-23 production (Supp Fig. 4). The remaining cytokines were not detected in either group suggesting that HIV infection of MDMs does not upregulate cytokines related to M1 phenotype. LCL161 treatment did not affect the secretion of the above-mentioned cytokines including CCL20/MIP3-α, IL-6, IL-23, IL-10, IL-21, IL-13, and TNF in *in vitro* HIV-infected MDMs (Supp Fig. 5A–G) or from *ex-vivo* derived MDMs from HIV-infected patients (Supp Fig. 5H). These results show that *in-vitro* HIV-infected MDM both before or after SM treatment did not express M1 phenotype and suggest that SM-mediated apoptosis of HIV-infected MDM is independent of M1 polarization.

### RIPK1 levels are reduced in HIV-infected MDMs

**Figure 7:**
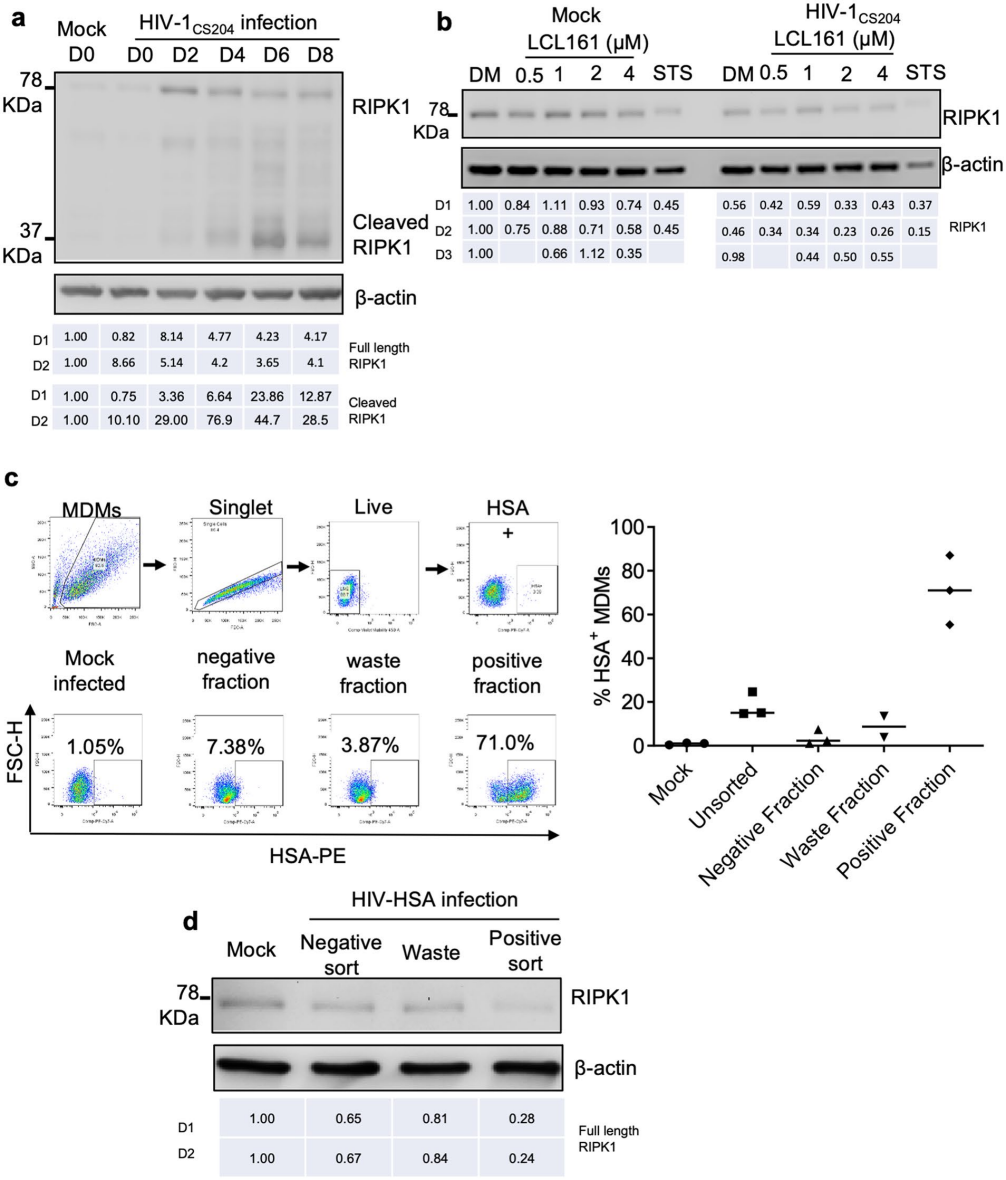
HIV infection results in downregulation of RIPK1 in MDMs. (**a**) MDMs were in vitro infected with HIV-1 CS204 (100 ng p24/well) and cells were harvested on day 0, 2, 4, 6, and 8. Cell lysates were subjected to western immunoblotting for RIPK1. The results shown are representative of 2 experiments. (**b**) MDMs were in vitro infected with HIV-CS204 (100 ng p24/well) for 7 days. Mock infected and HIV-1 CS204-infected MDMs were treated with different concentrations of LCL161 for 48 h. RIPK1 expression was assessed by western immunoblotting (n = 3). The uncropped western blot images are in Supplementary Fig. 8. (**c**) Gating strategy for detection of HIV-HSA-infected macrophages. HIV-infected bulk MDMs were gated as singlets followed by staining for live cells using e450 live/dead staining kit (Invitrogen). HIV-HSA-infected cells were detected within the live cell population by using FITC-labelled anti-CD24 antibodies (upper panel). MDM were in vitro infected with R5 tropic HIV-HSA as above for 11 days following which cells were subjected to magnetic column separation using CD24 (HSA)-biotin conjugated antibodies. The % of HIV-HSA-infected MDM in isolated unsorted, negative, waste and positive fractions as assessed by flow cytometry is shown (lower panel). (**d**) Isolated fractions of HIV-HSA, namely negative fraction (negative sort), waste fraction and positively isolated fraction (positive sort) were subjected to western immunoblotting for analysis of RIPK1 (n = 2). The uncropped western blot images are in Supplementary Fig. 9. The densitometric ratios are provided under the blots for (**a**, **b** and **d**) for different donors (D1, D2, D3).

SM-induced apoptosis of HIV-infected macrophages may be ascribed to the impaired expression of IAP-associated signaling kinases such as RIPK1^27,50^. RIPK1 plays a key role in NF-κB signaling and apoptosis^51^. Moreover, RIPK1 is a target substrate for HIV protease, a viral protein synthesized late in the viral life cycle that inactivate RIPK1 in HIV-infected primary CD4 + T cells^52^. Examining changes in RIPK1 over the course of MDM infection in vitro, we found an upregulation of full length RIPK1 at day 2 post-infection until day 8 post-infection (Fig. 7a) that was associated with a gradual and significant accumulation of cleaved RIPK1 on days 2, 4, 6 and 8 post-infections (Fig. 7a). The full length RIPK1 expression gradually declined following day 2 post-infection until day 8 post-infection (Fig. 7a). This suggested that in vitro infection of MDMs with HIV-1 CS204 resulted in the initial upregulation and subsequent down-regulation of full length RIPK1 and accumulation of cleaved RIPK1 over the period of HIV infection.

To determine whether RIPK1 is similarly cleaved and inactivated in HIV-infected MDMs treated with SM, in vitro mock and HIV CS204-infected MDMs (7 days post-infection) were treated with SM-LCL161 for 2 days followed by immunoprobing for RIPK1. HIV infection resulted in the downregulation of full length RIPK1 in the absence of LCL161 compared to the mock infected controls (densitometry ratio: compare DMSO lane 1 in both mock and HIV; Fig. 7b). SM-LCL161 treatment in both mock and HIV-infected MDM caused degradation of full length RIPK1 at all concentrations of LCL161 in both mock and HIV-infected macrophages (Fig. 7b). However, RIPK1 expression in HIV-infected LCL161-treated macrophages was lower compared to the mock-infected, LCL161-treated macrophages (Fig. 7b).

To confirm if the downregulation of RIPK1 occurs specifically in HIV-infected MDM, MDMs were infected with HIV-HSA for 9 days. HIV-infected HSA-expressing MDMs were harvested by magnetic column separation based on HSA expression^42^ followed by western blotting for RIPK1 analysis. The negative fraction represents HIV-exposed uninfected cells that do not express HSA on their surface, and hence get eluted after the first passing of the labelled cells. The waste fraction represents cells that are eluted during the column wash prior to the collection of the HSA-selected MDM and contains both HSA+ and HSA negative macrophages. The positive fraction represents the HIV-infected HSA-expressing cells retained in the magnetic column that are eluted at the end of the HSA selection protocol. The gating strategy for detection of HIV-HSA infected macrophages is shown (Fig. 7c upper panel). The positively selected MDM infected with HIV-HSA had ~ 70% purity while the negatively selected HIV-uninfected cells and waste fractions contained ~ 10 and ~ 5% HSA-expressing macrophages, respectively (Fig. 7c, lower panel). The blots show that full length RIPK1 is significantly downregulated in the positively selected HIV-HSA enriched fraction compared to the mock infected macrophages (densitometric ratio: 1.0 vs 0.28; Fig. 7d). Overall, the results suggest that full length RIPK1 is degraded with a corresponding increase in cleaved RIPK1 in HIV-infected macrophages.

### cIAP1/2 and RIPK1 are essential for survival of HIV-infected MDM

**Figure 8:**
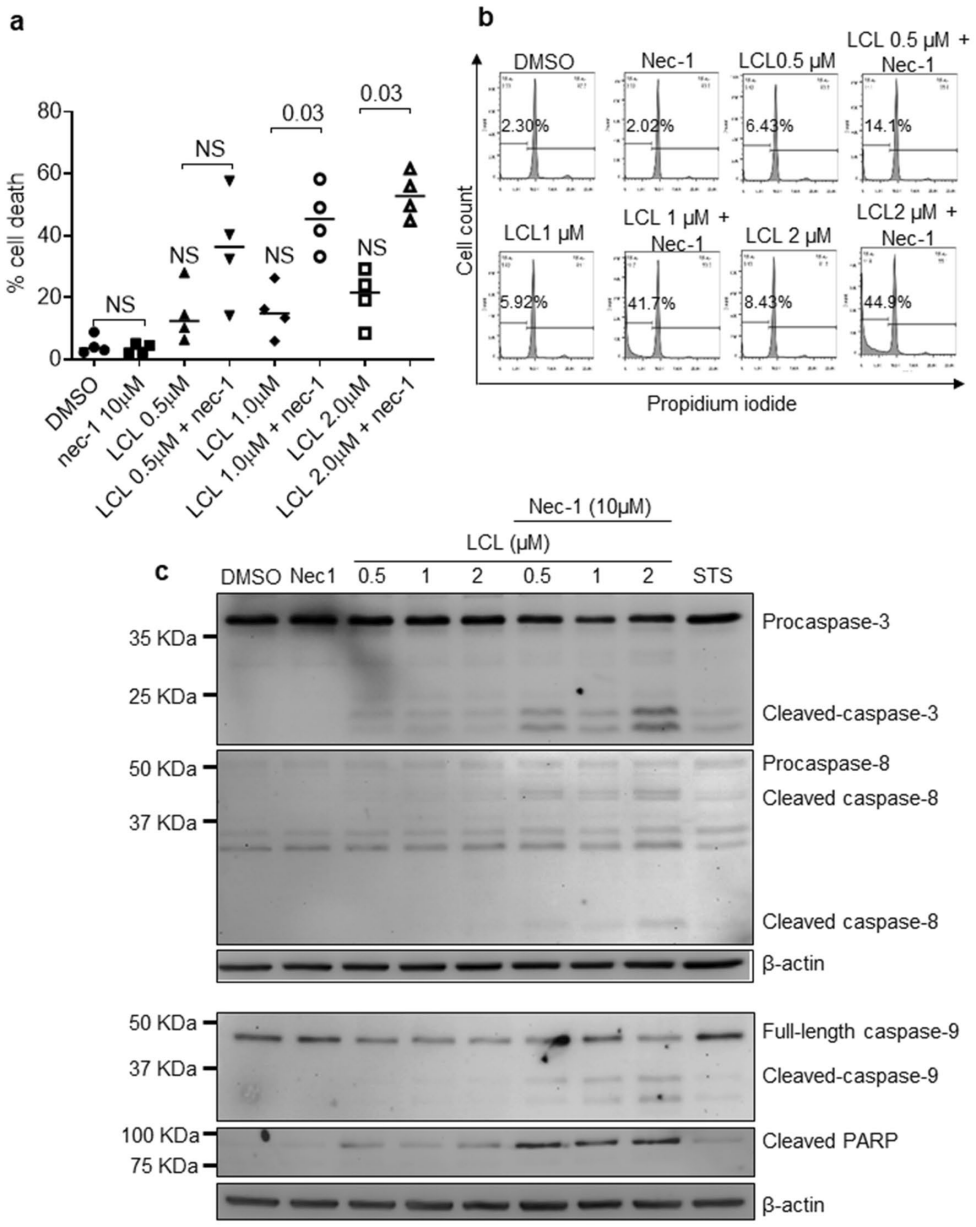
Concomitant downregulation of cIAP1/2 and RIPK1 in MDMs derived from healthy donors results in activation of apoptosis. (**a**) MDMs were treated with 10 μM necrostatin-1 for 2 h followed by the addition of increasing concentration of LCL161 for 48 h or staurosporin (STS). Cell death was assessed by intracellular PI staining and flow cytometry. The *p*-values were calculated using two tailed Mann–Whitney U test (n = 4). (**b**) The representative histograms of the four experiments are shown. (**c**) MDMs treated as above with necrostatin-1 and LCL161 were harvested and subjected to western immunoblotting for caspase-3, -8, and -9, cleaved PARP, and beta-actin. The blots shown are a representative of three experiments. The uncropped western blot images are in Supplementary Fig. 10. The densitometric readings for figure (**c**) are shown in Supplementary Fig. 11C.

The above results show that inactivation of RIPK1 in settings where cIAPs are absent, may affect the viability of macrophages. To evaluate the combined impact of cIAPs and RIPK1 inactivation on the survival of macrophages, MDM from healthy donors were pretreated with necrostatin-1 (nec-1), a specific RIPK1 inhibitor, for 2 h followed by treatment with LCL161 and analysis for cell death. Treatment with LCL161 or necrostatin-1 alone did not induce significant cell death in MDM. However, combined treatment of LCL161 and necrostatin-1 caused a significant increase in cell death of MDM (*p*-value = 0.03, LCL 2 μM *vs* LCL 2 μM + nec-1) (Fig. 8a). Figure 8b shows representative histograms of the intracellular PI staining. Furthermore, treatment with necrostatin-1 alone did not show cleavage of PARP or caspases-8 and 9 although treatment with LCL161 alone did show their minimal cleavage (Fig. 8c). However, treatment with both necrostatin-1 and LCL161 significantly enhanced cleavage of the three caspases as well as PARP (Fig. 8c). These results show that degradation of cIAP1/2 by LCL161 and inactivation of RIPK1 by necrostatin-1 results in death of normal macrophages and suggest that cIAP1/2 and RIPK1 play an important role in regulating viability of human macrophages. Since HIV infection down regulates RIPK1, and LCL161 causes IAPs degradation and death of HIV-infected macrophages, our results suggest that RIPK1 and IAPs may play key roles in SM-induced cell death of HIV-infected macrophages.

## Discussion

In this study, we show that although cIAP1/2 are dispensable host factors for the viability of macrophages, these anti-apoptotic proteins play a critical role in the survival of HIV-infected macrophages. SMs induced apoptosis of chronically HIV-infected U1 cells, in vitro HIV-infected macrophages with a clinical strain and *ex-vivo* derived macrophages from naïve and ART-treated HIV patients. Furthermore, SMs were shown to specifically kill HIV-infected MDMs by employing a R5-tropic laboratory strain, HIV-HSA. SM-induced killing of HIV-infected macrophages is mediated by apoptosis, but is independent of the establishment of M1 polarization. In addition, SM-induced apoptosis of HIV-infected macrophages may be due to RIPK1 degradation which in concert with IAP1/2 degradation may result in apoptosis of HIV-infected macrophages.

To achieve eradication of HIV-1 in patients undergoing suppressive ART, it is imperative to devise strategies to eliminate HIV reservoirs in cell targets other than T cells such as macrophages. Recently, cIAP1/2 and survivin, another member of the IAP family were suggested to be involved in survival of HIV-infected CD4 + T cells^30,31^ and macrophages^23^. In addition, XIAP down regulation by flavopiridol, a cyclin-dependent kinase 9 (CDK-9) inhibitor, caused increased apoptosis of a chronically HIV-infected ACH2 T cells^53^. We and others have previously shown that ablation of cIAP1/2 by SMs does not affect survival of primary human macrophages^36,54^. However, resistance of macrophages to apoptogenic HIV-Vpr was attributed to cIAP1/2^35–37^. These observations suggest that targeting of IAPs may represent a possible strategy for killing of HIV-infected macrophages. Herein, we show that in vitro HIV-infected macrophages with a clinical strain and macrophages generated ex vivo from ART-treated or naïve HIV-infected patients were susceptibility to SM-mediated cell death. For these studies, we employed the SMAC mimetics, LCL161 and AEG40730, to induce apoptosis in macrophages. LCL161 is a monovalent SM which binds IAPs with high affinity and initiates degradation of cIAP1 and cIAP2 (encoded by *BIRC2* and *BIRC3, * respectively) and prevention of caspase inhibition by XIAP^55^. AEG40730, on the other hand is a dimeric ATPF mimetic that activates caspase-8 to induce cell death. It is a bridged compound based on the caspase-9 amino terminus, which was linked via the threonine at the P2 position showing nM binding affinity for the BIR3 domains of XIAP, cIAP1, and cIAP2^27^. Both compounds have similar abilities to degrade IAPs. Further, the role of IAPs in mediating the induction of apoptosis was confirmed by using IAP siRNAs and two M-tropic strains, HIV-1 CS204 (clinical) and HIV-HSA. Our results agree with the observations made by Campbell et al. showing that LCL161 and birinapant selectively killed HIV-infected macrophages via autophagy-dependent apoptosis^23^. The role of autophagy in LCL161-induced cell death in our study needs to be investigated.

Our results on the effect of SM on macrophages derived from the HIV-infected patients show killing of 20–30% macrophages. However, HIV-infected myeloid cells are rarely detected in the blood of HIV-infected individuals^41^; hence the frequency of HIV-infected macrophages in ex vivo derived macrophages from HIV-infected patients may be extremely low. Therefore, it is likely that SMs may be killing bystander cells (Fig. 2f, g) unlike the in vitro HIV-HSA-infected macrophages. It is extremely difficult to determine selective killing of very low numbers of HIV-infected macrophages in the patients and even in in vitro HIV-infected macrophages. The high variability in SM-mediated killing of ex vivo generated MDMs from monocytes of ART-treated and naïve untreated groups may also be due to the myeloid population in the patients which is exposed to a variety of inflammatory/anti-inflammatory cytokines and exposure to a variety of opportunistic infections^56^. Exposure to ARV drugs may be somewhat toxic to these cells and affect their ability to respond to stress by altering their mitochondrial function^57,58^.

The number of HIV-infected macrophages in in vitro infected macrophages with the clinical strain is around 5–10% and varies up to 30% with HIV-HSA due partly to a milieu of HIV restriction factors that limit the virus life cycle^12,42^. Although SMs killed high number of HIV-HSA-negative macrophages compared to mock-infected macrophages, the differences were not significant. Moreover, SMs killed significantly higher number of HIV-HSA-infected macrophages compared to the HIV-HSA-negative macrophages further suggesting a specificity of SMs towards killing of HIV-infected macrophages. We did not observe a drop in the percentage of infected cells following exposure to SMs as Annexin-V detects early stage of apoptosis. These Annexin positive cells we observed should eventually die at a later stage. Consistent with this, we did observe a significant drop in the percentage of infected cells after 7 days of exposure to SMs.

IAPs have been shown as a potent negative regulator of LTR-dependent HIV-1 transcription causing reversal of HIV latency in JLat latency model system and in latently infected memory T cells^32^. SMs activate the non-canonical NF-κB pathway by virtue of RelA:p50 and RelB:p52 transcription factors, which bind to the HIV-1 LTR region leading to the induction of virus transcription in latently infected JLat cells^32,59^. However, SM did not affect HIV transcription in U1 cells and in vitro HIV-infected macrophages. Our results are also in agreement with Campbell et al. on the effects of SM on HIV transcription in HIV-infected macrophages^23^. We have previously shown that SMs alone did not activate either classical or alternative NF-κB pathways in macrophages^40^, which may explain SM’s inability to impact virus replication in macrophages.

SM-mediated killing has been attributed to endogenous TNF in cancer cells^45,46^; however, it has been reported to be independent of TNF in some cancer cells^60^ and HIV-infected T cells^32^. While TNF-mediated SM-induced killing in U1 undifferentiated and PMA-differentiated cells, it was not observed in primary MDMs. Although in vitro HIV-infected MDMs produced significant levels of TNF, SM treatment did not affect its secretion in either uninfected or HIV-infected macrophages. Moreover, rTNF did not induce cell death in SM-treated macrophages suggesting that SM-induced cell death in macrophages is independent of TNF. We have also shown that SM-induced apoptosis in M1 macrophages was not mediated by TNF although M1 macrophages produced high levels of TNF^61^. However, the involvement of TNF in SM-induced cell death cannot be conclusively ruled out without silencing of TNF receptors in HIV-infected macrophages.

HIV infection results in dysregulation of cytokine profile in-vivo and in-vitro^62^ and can possibly affect the polarization state of macrophages**.** Since IFN-γ-generated M1 macrophages are susceptible to SM-mediated cell death^49^, HIV-1 infection may polarize macrophages into M1 phenotype and make them susceptible to SM-induced apoptosis. Since in vitro-infected and ex vivo*-* derived macrophages exposed to SM were not polarized into M1 phenotype suggested that SM-mediated killing of HIV-infected macrophages was not due to M1 polarization.

The pathways of apoptosis are regulated by RIPK1^51,63^. In TNF-mediated signaling, RIPK1 is recruited in a multiprotein complex I along with TRADD, TRAF2, and cIAP1/2 to promote transcription of genes with anti-apoptotic properties such as cIAP1/2^63^. RIPK1 is also recruited in a protein complex composed of TRADD, FADD, and caspase-8, which depending on additional proteins recruited, can induce apoptosis or necroptosis^63^. Recently, HIV infection of primary activated CD4^+^ T cells was shown to downregulate RIPK1 through the HIV-1 protease^52^. RIPK1 modification in response to human rhinovirus and Newcastle disease virus infection has also been reported^64,65^. Herein, we show that infection of macrophages with HIV-1 CS204 or with HIV-HSA caused downregulation and cleavage of RIPK1. Given that down regulation of IAPs alone by LCL161 or of inactivation of RIPK1 alone by necrostatin-1 did not induce cell death in uninfected macrophages, suggests that RIPK1 and cIAP1/2 are dispensable in survival of macrophages. However, inactivation of RIPK1 by necrostatin-1 following IAP degradation by SM resulted in a dramatic increase in cell death, cleavage of caspases and PARP in normal macrophages suggesting that RIPK1 may play a key role in SM-induced killing of HIV-infected macrophages. The role of RIPK1 degradation during HIV-1 infection of macrophages needs further investigation.

In summary, the results of this study suggest a potential significance of SM in killing of HIV-infected macrophages in vivo. In support of these observations, recent reports using the combination of an LRA and SMAC mimetic^66^, and nanoengineered CD4 + T cell membrane-coated nanoparticles (TNP) encapsulating the SMAC mimetics LCL161 have been shown to be potential therapeutic agents to kill specifically HIV-1-infected cells as part of an HIV-1 cure strategy^67^. In the event SM are able to kill HIV-infected macrophages in vivo*,* they have the potential to be translated into clinical interventions aimed at eradicating HIV infection by directly targeting HIV-infected macrophages.

## Methods

### Generation of human MDMs, cell lines and reagents

Human peripheral blood mononuclear cells (PBMCs) were isolated by density gradient centrifugation using Ficoll Paque (GE Healthcare Life Sciences Buckingmshire, UK) from the blood of healthy donors. MDMs were generated from monocytes via adherence methods as previously described^40^. Briefly, 2.0 × 10^6^ PBMCs/well (12-well plate) were allowed to adhere for 3 h and non-adherent cells were washed off. Adherent monocytes were cultured for 7 days in complete DMEM (Wisent Inc., St. Bruno, Quebec) supplemented with 10% fetal bovine serum (FBS; GE Healthcare), penicillin and streptomycin and 10 ng/ml MCSF (R&D Systems, Minneapolis, MN, USA). MCSF-containing media was replaced every 2 days until the 7th day at which point the monocytes differentiated into macrophages. Purity of macrophages as assessed by measuring CD14 expression by flow cytometry was 100%. Using a similar protocol, MDMs were generated from PBMCs obtained from HIV-infected individuals receiving anti-retroviral treatment (typically one integrase strand transfer inhibitor plus two nucleosides/nucleotide analogues) and from HIV-infected individuals who never received any treatment (ART naïve).

U937 (NIH-ARP Cat# 127-109, RRID:CVCL_X608) and U1 (NIH-ARP Cat# 165-432, RRID:CVCL_M769) cells were obtained from NIH AIDS reagent program and were cultured in complete DMEM media. U937 is a leukemic pro-monocytic cell line whereas U1 is a subclone of U937 chronically infected with HIV-1. U1 cells carry two copies of proviral DNA and show its minimal constitutive expression. Differentiation with PMA (Sigma Aldrich, St. Louis, Missouri, USA) induces virus expression in these cells^38^. For differentiation, 5.0 × 10^5^ U937 and U1 cells were treated with 20 nM PMA (Sigma Aldrich) for 2 days. LCL161 (APExBIO, Houston, Texas, USA), AEG40730 (Bio-Techne, Toronto Canada), necrostatin-1 (APExBIO, Houston, Texas, USA), staurosporine (STS) (both from APExBIO, Houston, Texas, USA), and LPS (Sigma Aldrich) were purchased.

### HIV-1 production, in vitro infection of MDMs with HIV and HIV p24 ELISA

MDM were infected with dual tropic HIV-1 CS204 viral stock supernatants containing 100 ng of HIV-p24 protein, supplemented with 8 µg/ml of polybrene (Sigma-Aldrich) for 2 h as described previously^68^. The dual tropic HIV-1 CS204 stocks were produced in CD8^+^ depleted PBMCs from healthy donors as previously described^69^. Stocks of mock virus were produced under similar conditions but in the absence of HIV. Following infection, MDM were washed three times with DPBS and cultured in complete medium. Supernatants were collected every 3 to 4 days for determining productive infection of MDM by HIV-p24 ELISA. Aliquots of the mock and viral stocks or supernatants were inactivated for 1 h at 37 °C in 1% Triton X-100 (Sigma-Aldrich). These experiments utilized reagents provided by the AIDS and Cancer Virus Program, Leidos Biomedical Research, Inc., Frederick National laboratory for Cancer Research, supported with federal funds from the National Cancer Insitute, National Institute of Health, under contract HHSN261200800001E.

The plasmid pNL4.3-Bal-IRES-HSA (provided by Dr M. Tremblay, Laval University, Quebec, Canada) was amplified using One Shot® Stbl3™ competent *E. coli* **(**Invitrogen, Carlsbad, CA, USA) and isolated using endotoxin-free plasmid DNA isolation mega kit (Thermo Fisher Scientific). To produce stocks of HIV-HSA (NL4.3-Bal-IRES-HSA) and mock viruses, 50 µg endotoxin-free plasmid DNA were transfected into 293 T cells with 125 µl of Lipofectamine™ 2,000 (Invitrogen) at a density of 5 × 10^6^ cells/10 cm dish. The supernatants harvested at 48 and 72 h were combined and centrifuged at 2000 g for 15 min. PEG-it™ virus precipitation solution (SBI, Biotech, Japan) was used to precipitate viruses at 4 °C for 24 ~ 48 h and centrifuged at 2,000 g for 30 min. The precipitates were resuspended in DPBS with 0.05 M HEPES and stored at − 80 °C. Viruses were quantified by HIV p24 ELISA as above.

MDMs were infected with 100 ng p24 per 1.0 × 10^6^ cells supplemented with 5 μg/ml polybrene (Sigma Aldrich SKU: TR-1003-G) for 16 h followed by infection with HIV-1 CS204 or HIV-HSA for 7 days. The supernatants were assessed for p24 using HIV p24^CA^ ELISA capture kit as above.

### Treatment of MDM with SM and assessment of apoptosis by flow cytometry

MDMs were cultured in complete media without antibiotics for 2 h before treatment with various concentrations of AEG40730 or LCL161. MDMs were evaluated for cell death by using intracellular PI staining as described^35,40^. Briefly, cells were washed with DPBS and fixed with methanol for 15 min at 4 °C. Subsequently, cells were treated with 25 μl of 10 μg/ml RNase A, followed by staining with 25 μl of 1 mg/ml PI solution (Sigma-Aldrich) at 4 °C for 1 h. The DNA content was analyzed using a Fortessa X-20 cytometer (BD Biosciences, Franklin Lakes, NJ, USA) and the FlowJo v10 software. The subdiploid DNA peak (< 2 N DNA), immediately adjacent to the G_0_/G_1_peak (2 N DNA), represents apoptotic cells and was quantified by histogram analyses.

### Quantification of apoptosis in HIV-HSA-infected MDMs

For quantification of apoptosis in HIV-HSA-infected MDMs following treatment with either SM or siRNA transfection, cells were harvested after trypsinization with 0.25% Trypsin–EDTA (Gibco, Dublin, Ireland) for 30 min. HIV-HSA-infected MDMs were blocked with FcR blocking reagent (MACS Miltenyi Biotec, Auburn, CA, USA) in 50 µl of 5 g/L BSA in DPBS, and stained with 2.0 µl of FITC conjugated anti-mouse CD24 antibody (BD Biosciences) for 20 min. HIV-HSA-infected MDMs were stained with 2.0 µl Annexin-V-BV711 (BD Biosciences) in 50 µl of 5 g/L BSA in DPBS for 15 min. Cells were subsequently washed and fixed with 1% PFA (Affymetrix, Santa Clara, CA, USA) followed by analysis with Fortessa X-20 cytometer at FITC and BV711 channels and FlowJo v10 software.

### siRNA transfection of MDMs

Transfection of MDMs with siRNAs was performed as described earlier^70^. Briefly, 20 nM of siRNA mixture (XIAP, cIAP1 and cIAP2) was added to 200 µl of Opti-MEM™ I Reduced Serum Medium with 1.0 µl DharmaFect 3 (Dharmacon, Colorado, USA). Using non-targeting (NT) siRNA-Alexa Fluo555 and DharmaFect 3 transfection reagent (B2), ~ 85% transfection efficiency was achieved in MDMs^70^.

### Isolation of HIV-HSA-infected MDMs

MDMs were infected with HIV-HSA for 9 days followed by magnetic sorting using HSA-CD24 beads (MACS Miltenyi Biotec.) through column separation as previously described^42^. Briefly, infected MDMs were detached with accutase (Innovative Cell Technologies, San Diego, CA), FcRγII receptors were blocked with FcR blocker (cat# 130-059-901 MACS Miltenyi Biotec), stained with primary CD24-biotin conjugated antibody and incubated with anti-biotin ultra-pure microbeads (cat#130-098-902 MACS Miltenyi Biotec). HSA-expressing cells were collected by positive selection in LS columns. The HSA-negative fraction was collected after passing the labelled cells through the column for the first time. The column was detached from the magnet and the HSA-positive cells were collected by plunging out the cells. Purity of the HIV-HSA-infected macrophages was assessed by flow cytometry using anti-Biotin PECy7 antibody.

### Analysis of caspase activation

Activation of caspase-3 (cat# 270771), -8 (cat# 65614), and -9 (cat# ab61615) was measured as per Abcam’s Caspase staining kit protocol (Abcam, Toronto, Ontario, Canada) by flow cytometry.

### Quantification of total HIV-DNA and integrated HIV-DNA by ultrasensitive qPCR

Total HIV-DNA and integrated HIV-DNA were quantified using nested ultrasensitive PCR previously described^26,71^. Briefly, HIV-infected MDMs were harvested and ACH2 cells were lysed overnight in a thermoshaker using lysis buffer [(Proteinase K (Invitrogen, Carslbad, California, USA cat # 25530-015), 0.1 M Tris HCl pH8, 0.5 M KCl)] overnight at 55 C, 450 rpm. PKC was inactivated by heating at 95 C for 10 min. 15 µl of the lysate was used in the first round of amplification in nested HIV-DNA qPCR. The primer sets used for the first round of amplification of total HIV-DNA were ULF1 and UR1, while ULF1, Alu1, and Alu2 were used for integrated HIV-DNA (ULF1: 5′-ATG CCA CGT AAG CGA AAC TCT GGG TCT CTC TDG TTA GAC-3′, UR1: 5′-CCA TCT CTC TCC TTC TAG C-3′, Alu1: 5′-TCC CAG CTA CTG GGG AGG CTG AGG-3′, Alu2: 5′-GCC TCC CAA AGT GCT GGG ATT ACA G-3). Primer sets for human CD3 gene (HCD3OUT5′ and HCD3OUT3′) were used to quantify the exact number of cells per reaction mixtures in all PCRs. The first PCR products were diluted 10X and 6.8 µL was subjected to second round of amplification in two different reaction mixtures using primers sets HCD3in5′: 5′-GGC TAT CAT TCT TCT TCA AGG T-3′ and HCD3 in 3′: 5′-CCT CTC TTC AGC CAT TTA AGT A-3′ for human CD3 quantification, and LambdaT: 5′-ATG CCA CGT AAG CGA AAC T-3′ and UR2: 5′-CTG AGG GAT CTC TAG TTA CC-3′ for HIV quantification. CD3 FamZen: 5′-/56-FAM/AG CAG AGA A/ZEN/C AGT TAA GAG CCT CCA T/3IABkFQ/-3′ and UHIV FamZen: 5′-/56-FAM/CA CTC AAG G/ZEN/C AAG CTT TAT TGA GGC /3IABkFQ/-3′ probes were added separately to each reaction mixture. Known HIV and CD3 copy numbers of ACH2 cells were used to generate a standard curve for absolute quantification of HIV DNA per million cells.

### TNF ELISA

Human TNF duo set (R&D System) was used to quantify TNF as per the manufacturer recommendations. Briefly, 96-well plates were preincubated with TNF capture antibody for 16 h followed by blocking with 1% FBS. TNF (1–1000 pg/ml) was used as standards. The samples were added to the plates for 16 h followed by the detection antibodies for another two h. Next, 100 µL/well of substrate solution was added. The enzymatic reaction was stopped with 50 μL/well of stop solution (BioFX Labs, Owing Mills, MD). The plates were read at 490 nM using iMark Microplate reader (Biorad, Mississauga, Ontario) using microplate manager 6 software.

### Cytokine ELISA array

The levels of secreted cytokines were measured as per the directions in Milliplex map kit (Millipore, Etobicoke, ON, Canada). IL-17F, GM-CSF, IFNγ, IL-10, CCL20/MIP3α, IL-12p70, IL-13, IL-15, IL-17, IL-22, IL-9, IL-1β, IL-33, IL-21, IL-23, IL-5, IL-6, IL-17ε/IL-25, IL-27, IL-31, TNFα, TNFβ, and IL-28A were detected using antibody-immobilized magnetic beads and were quantified by MAGPIX® multiplex with xPONENT® software (Luminex Corp.).

### Western immunoblot analysis

The lysates were subjected to SDS-PAGE electrophoresis as described earlier^35,40,72^. Proteins were transferred onto polyvinylidene difluoride membrane (BioRad Laboratory, Hercules, CA) and blocked with 20 g/L BSA or 1% skim milk solution for 1 h. Membranes were then washed 3X with TBST buffer and thereafter probed with primary antibodies specific for cIAP1 (clone D5G9 1:1000 dilution, cat# 7065, RRID:AB_10890862), cIAP2 (clone 58C7 1:1,000 dilution, cat# 3130S, RRID:AB_10693298), caspase-3 (1:500 dilution, cat# 9662, RRID:AB_331439), caspase-8 (1:500 dilution, cat# 9496, RRID:AB_561381), caspase-9 (1:500 dilution, cat# 9502)., RRID:AB_2068621), β-actin (clone13E5, 1:1,000 dilution, cat # 4970, RRID:AB_2223172), PARP (1:1,000 dilution, cat#9542, RRID:AB_2160739), RIPK1 (clone D94C12, 1:1,000 dilution, cat # 3493 (Cell Signaling Tech, Inc., Danvers, MA), followed by goat anti-rabbit or anti-mouse secondary polyclonal antibodies conjugated to horseradish peroxidase (BioRad Laboratory). Proteins were visualized by enhanced chemiluminescence (Cytiva, Global Life Sciences Solutions Canada, British Columbia, Canada).

### Statistical analysis

Data was plotted using Graphpad Prism 10. The statistical significance was calculated using student paired t test or Mann–Whitney U test. Plotted data represent the mean ± SD.

### Ethics statement

HIV-infected individuals and healthy participants involved in the study gave informed written consent and the protocol for obtaining blood samples was approved by the Review Ethics Board of the Ottawa General Hospital and the Children’s Hospital of Eastern Ontario, Ottawa, ON, Canada. All methods were performed in accordance with relevant guidelines and regulations.

## Supplementary Information

Supplementary Figures.
