# Author Correction: Role of RIPK1 in SMAC mimetics-induced apoptosis in primary human HIV-infected macrophages

**DOI:** 10.1038/s41598-021-03517-z

**Published:** 2021-12-08

**Authors:** Ramon Edwin Caballero, Simon Xin Min Dong, Niranjala Gajanayaka, Hamza Ali, Edana Cassol, William D. Cameron, Robert Korneluk, Michel J. Tremblay, Jonathan B. Angel, Ashok Kumar

**Affiliations:** 1 Department of Biochemistry, Microbiology, and Immunology, Faculty of Medicine, University of Ottawa, Ottawa, ON Canada; 2 Division of Virology, Apoptosis Research Centre, Children’s Hospital of Eastern Ontario Research Institute, 401 Smyth Road, Research Building 2, University of Ottawa, Ottawa, ON K1H 8L1 Canada; 3 Department of Health Sciences, Carleton University, Ottawa, ON Canada; 4 Division of Infectious Diseases, The Ottawa Hospital Research Institute, Ottawa, ON Canada; 5 Centre de recherche du CHU de Québec‑Université Laval, Université Laval, Québec City, QC Canada; 6 Department of Pathology and Laboratory Medicine, Faculty of Medicine, University of Ottawa, Ottawa, ON Canada

Correction to: *Scientific Reports* 10.1038/s41598-021-02146-w, published online 25 November 2021

The original version of this Article contained an error in the spelling of the author Jonathan B Angel which was incorrectly given as Jonathan Angel.

The original Article has been corrected.

