# Supplementary material for: Role of RIPK1 in SMAC mimetics-induced apoptosis in primary human HIV-infected macrophages
Source: Sci Rep. 2021 Nov 25;11:22901. doi: 10.1038/s41598-021-02146-w (PMC8617210; doi:10.1038/s41598-021-02146-w)
Supplement: Supplementary file 1 — Supplementary Figures. [file 41598_2021_2146_MOESM1_ESM.pdf]

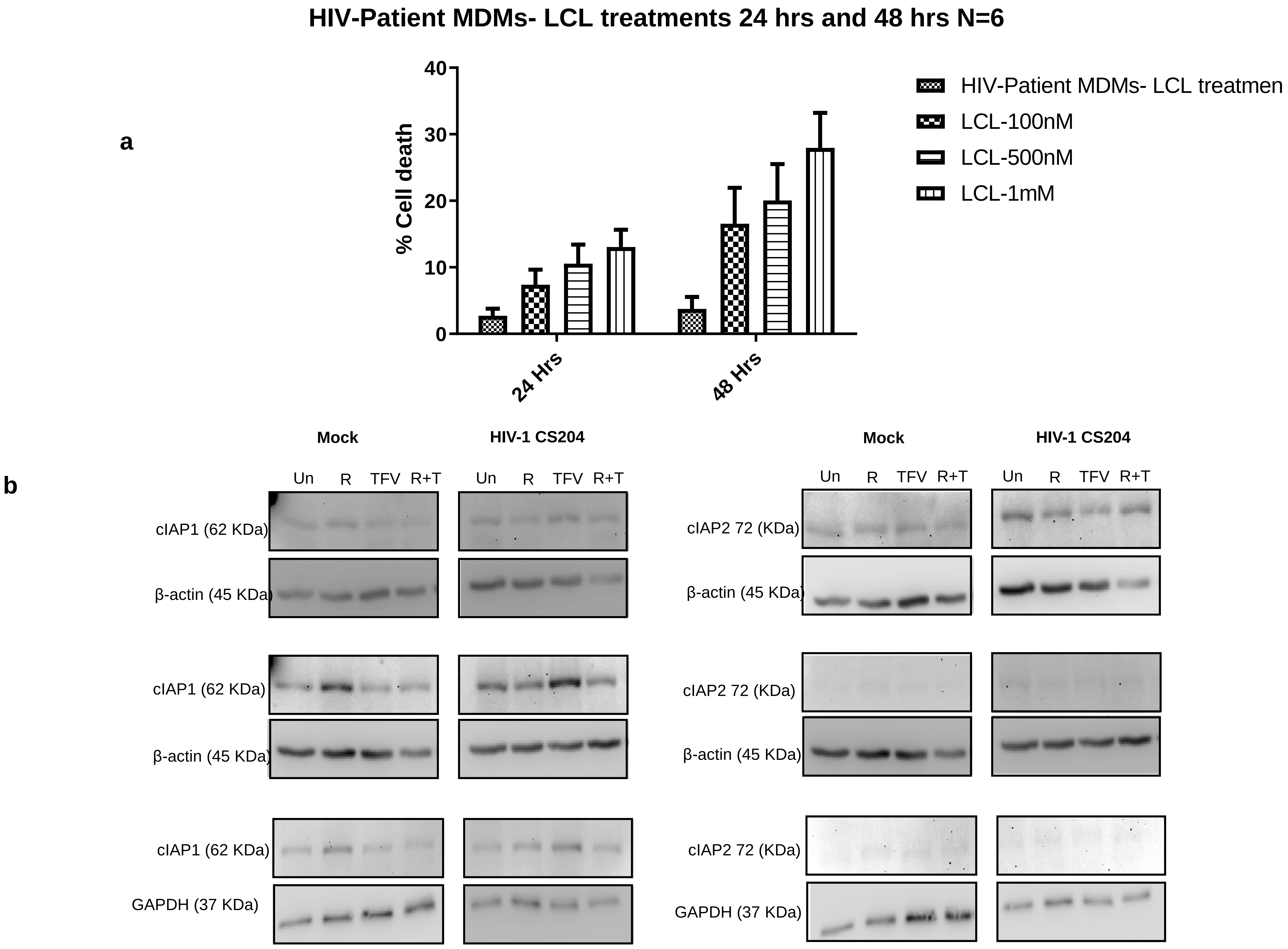

Suppl. Fig 1. (A) MDMs generated from naïve and ART-treated HIV-individuals were treated with various concentrations of SM-LCL161 for 24 and 48 hr. Cell death was assessed by PI staining and flow cytometry. (B) MDMs were treated with the indicated concentrations of Smac mimetics LN or SM for 24 h. Cells were collected and analyzed for IAPs protein expression by Western blotting. (B) Antiretroviral treatment of mock and HIV-infected MDMs did not affect the expression of cIAP1 and cIAP2. MDMs were *in vitro* infected with mock and HIV-1 CS204 for 7 days followed by antiretroviral drugs (Raltegravir (1 μg/mL) = R, Tenofovir (10 μM) = TFV) treatment for 48 hours. The cells were lysed and levels of cIAP1 and cIAP2 and loading controls β-actin and GAPDH were analyzed by western immunoblotting. The uncropped original blots for figures 1B, donors 1, 2 and 3 are shown in supplementary Figures 12a, b, c, d, e and f..

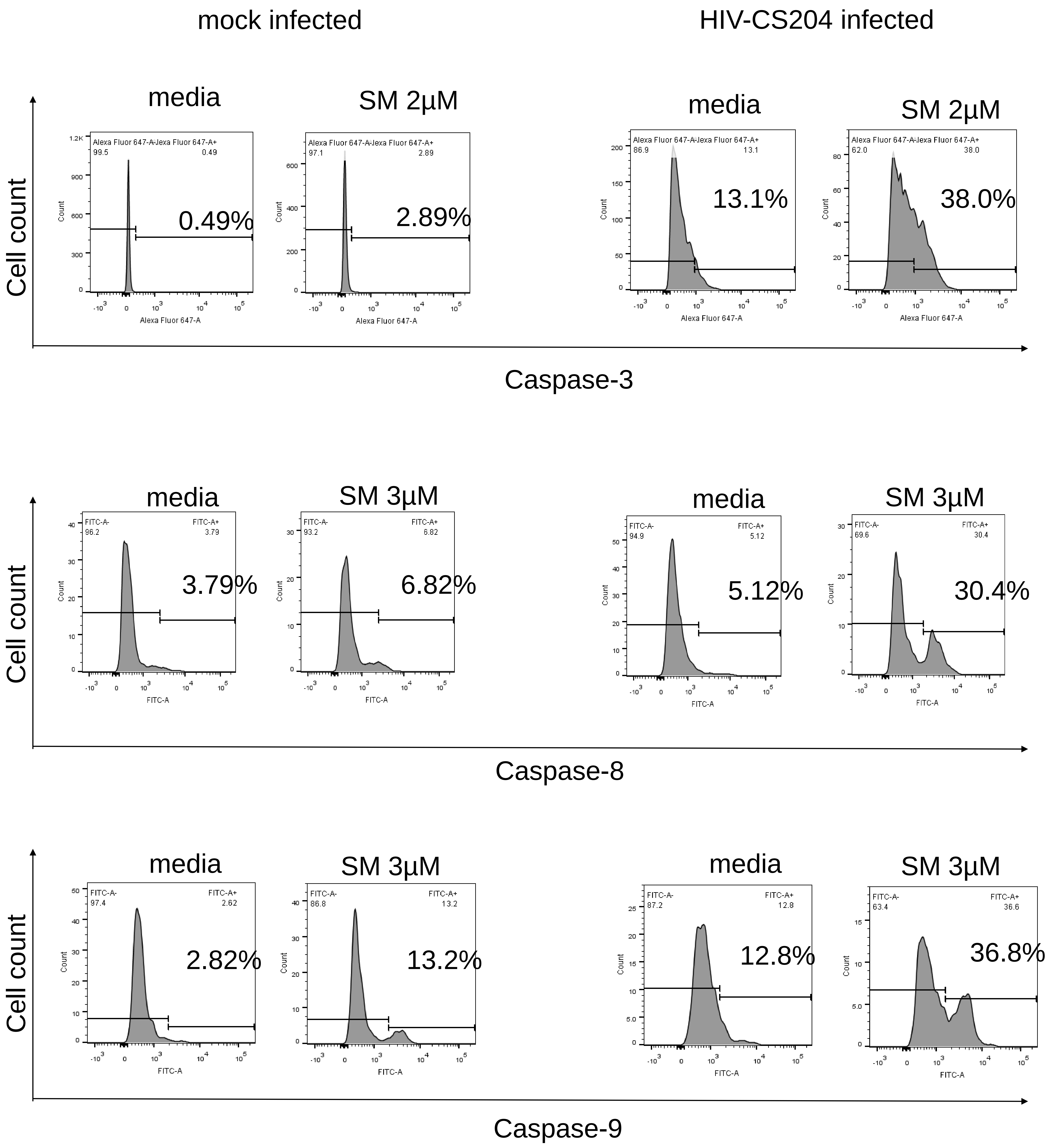

Supp Fig 2. SM induce the activation of caspases in HIV-infected MDMs. Human MDMs were in vitro infected with HIV-1 CS204 (100 ng p24 / well) for 7 days. The cells were then treated with LCL161 for 48 hr. The activation of the caspase-3, 8, and 9 were detected by intracellular caspases staining and flow cytometry. Representative histograms are shown.

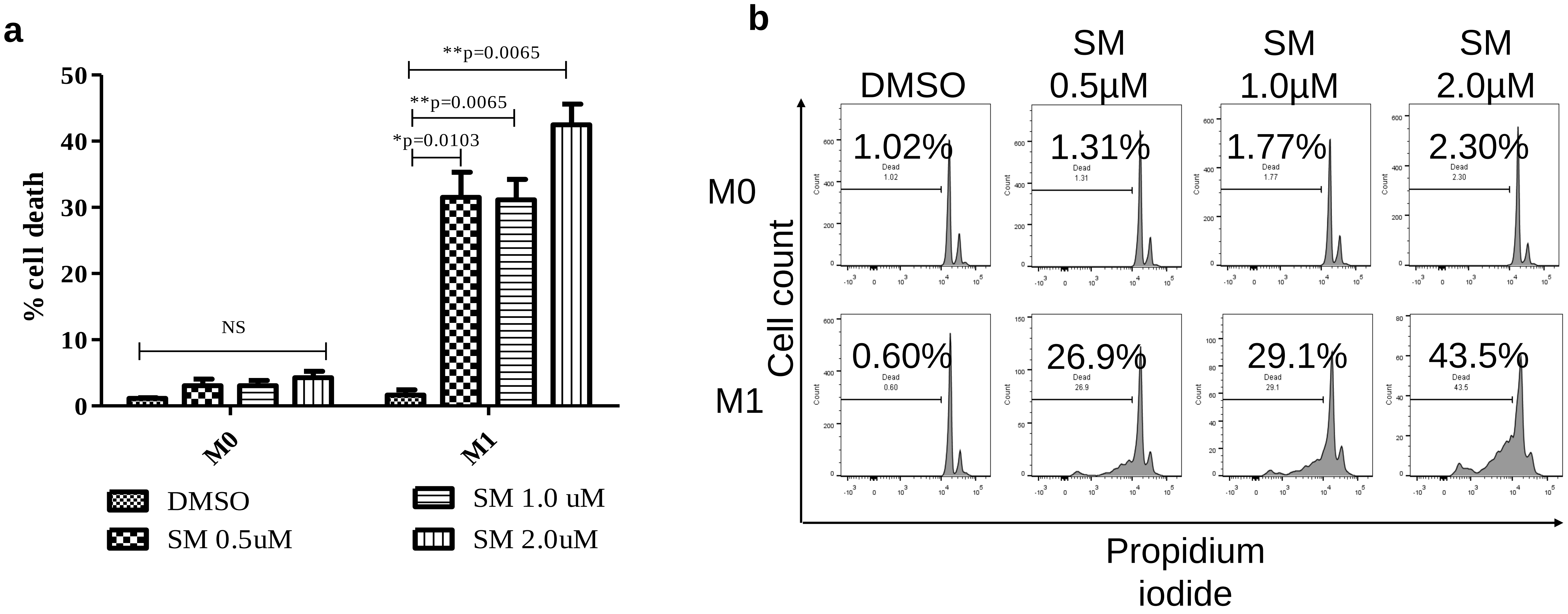

Supp Fig 3. SMs induce cell death in M1 macrophages. (A). M0 and M1 MDMs were treated with increasing concentration of LCL161 48 hr (n=3). Cell death was assessed by intracellular PI staining and flow cytometry. The p-values were calculated using two-tailed Mann-Whitney U test. (B) A representative histograms for cell death in M1 macrophages is shown.

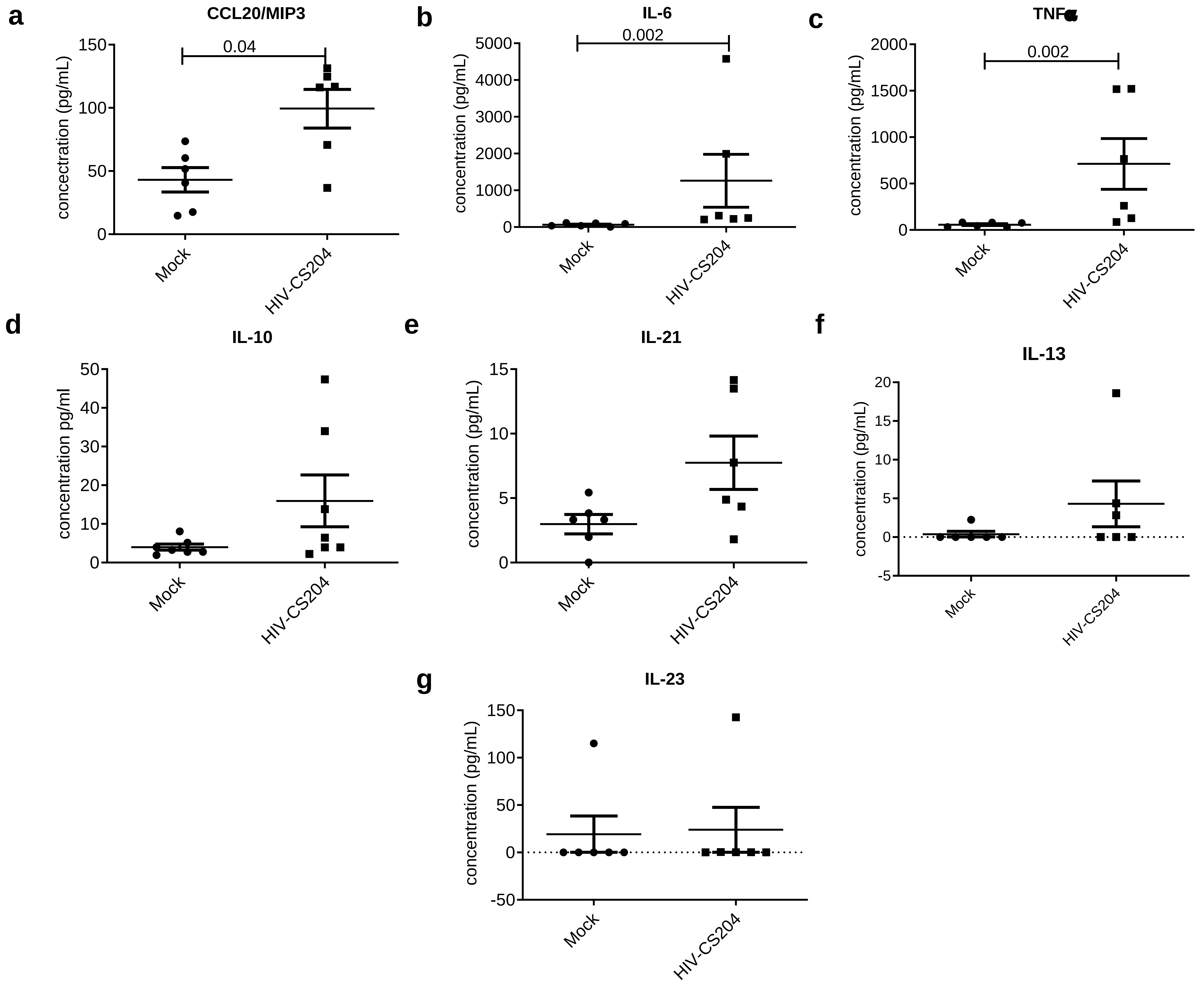

Suppl. Fig 4. HIV infection of MDMs does not result in the upregulation of cytokines related to M1 phenotype. MDMs were *in vitro* infected with mock or HIV-1 CS204. The supernatants collected after 7 days of infection were analyzed for the secretion of cytokines using Human Th17 magnetic panel cytokine array kit for 22 different cytokines (n=6). The p-values were calculated using two-tailed Mann-Whitney U test

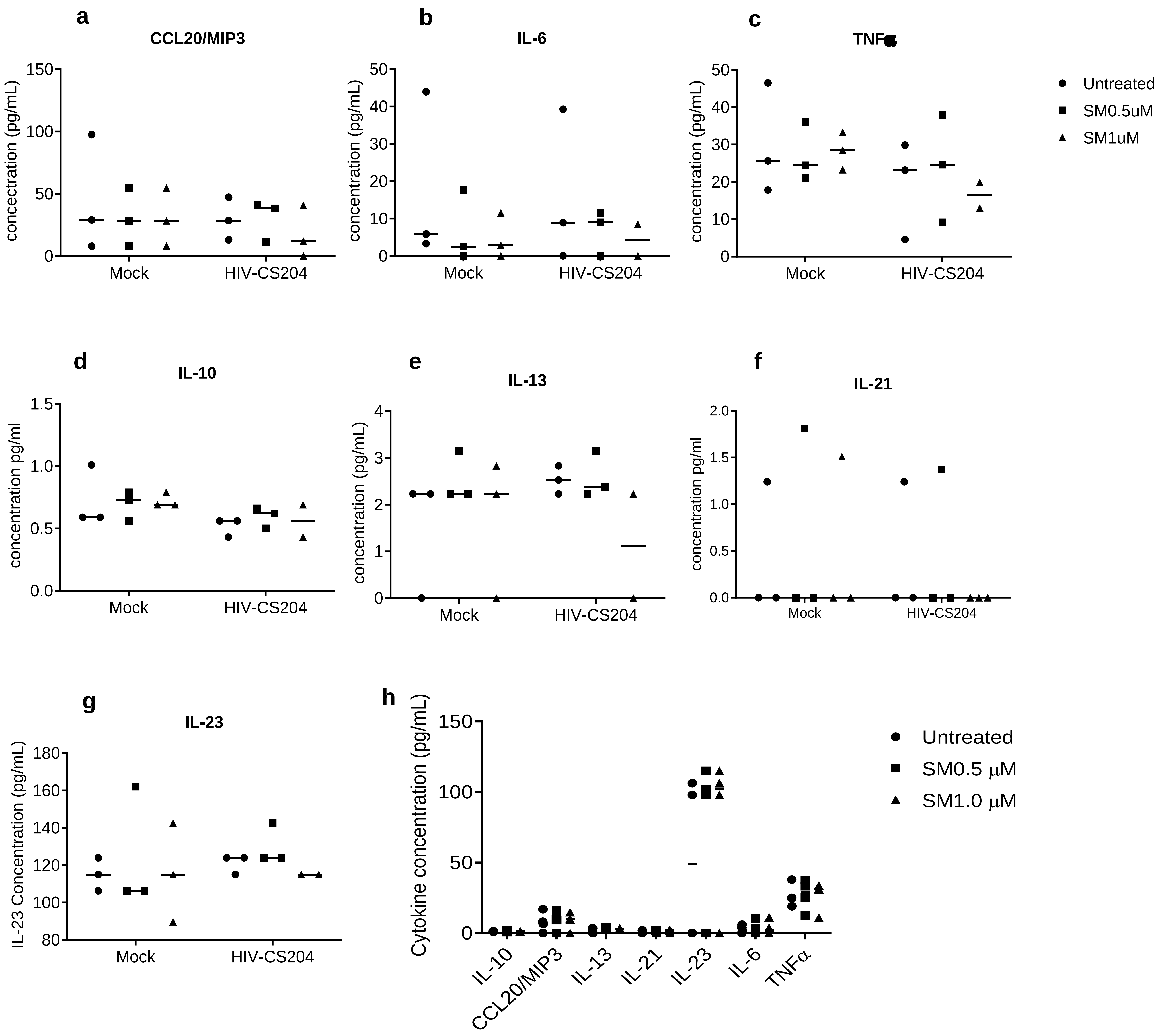

Suppl. Fig 5. (A-G) SM does not induce aberrant production of M1 cytokines in mock and HIV-infected MDMs. The *in vitro* mock and HIVcs204-infected MDM for 7 days were treated with SM LCL161 for 48 hr. Supernatants were collected, and cytokine profile was analyzed through Human Th17 magnetic panel cytokine array kit (n=3) (H). SM does not induce cytokine production in MDMs generated from HIV-infected individuals. PBMC from ART-treated HIV+ patients were differentiated into macrophages for 7-days and subsequently treated with SM LCL161 for 48 hr. The supernatants were analyzed for cytokines through Human Th17 magnetic panel cytokine array. P-values were calculated using paired-T test (n=3)

Fig 1C

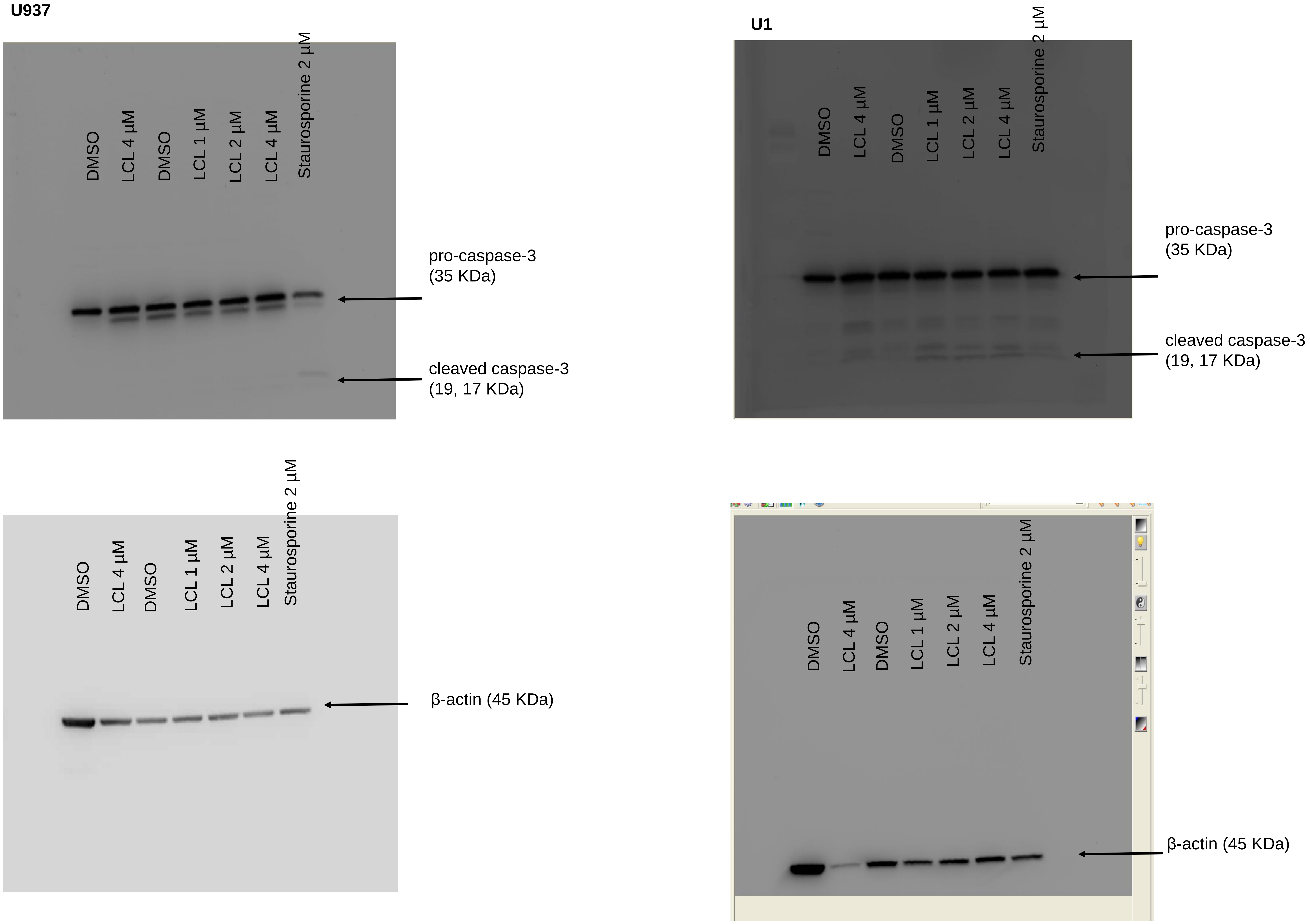

Suppl.Fig 6. Full blots for Fig 1C Caspase-3 and beta-actin for U937 and U1 cells.

Fig 2A

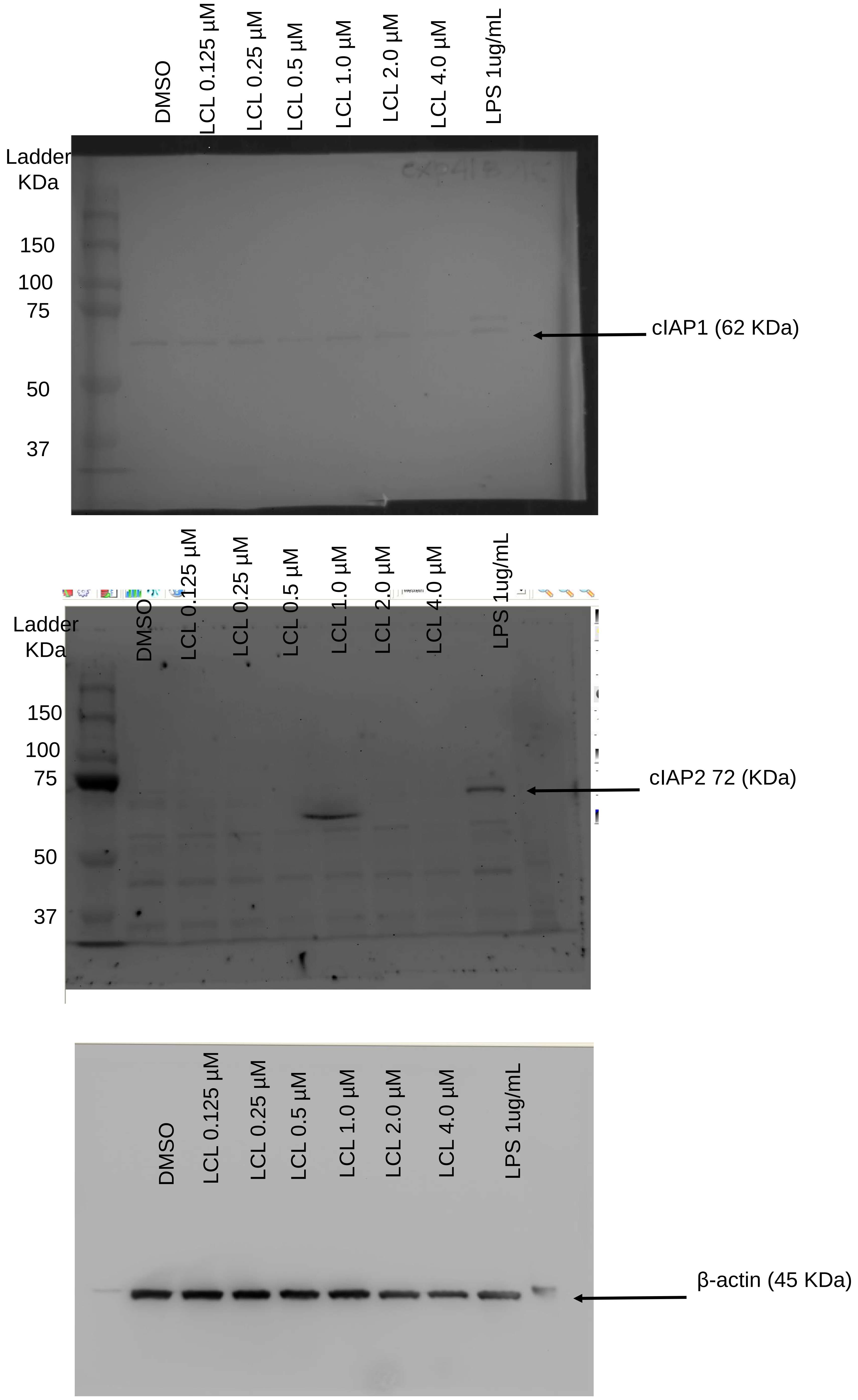

Suppl. Fig 7. Full blots for Fig2A cIAP1 and cIAP2 degradation by LCL161 in MDMs..

Fig 7A

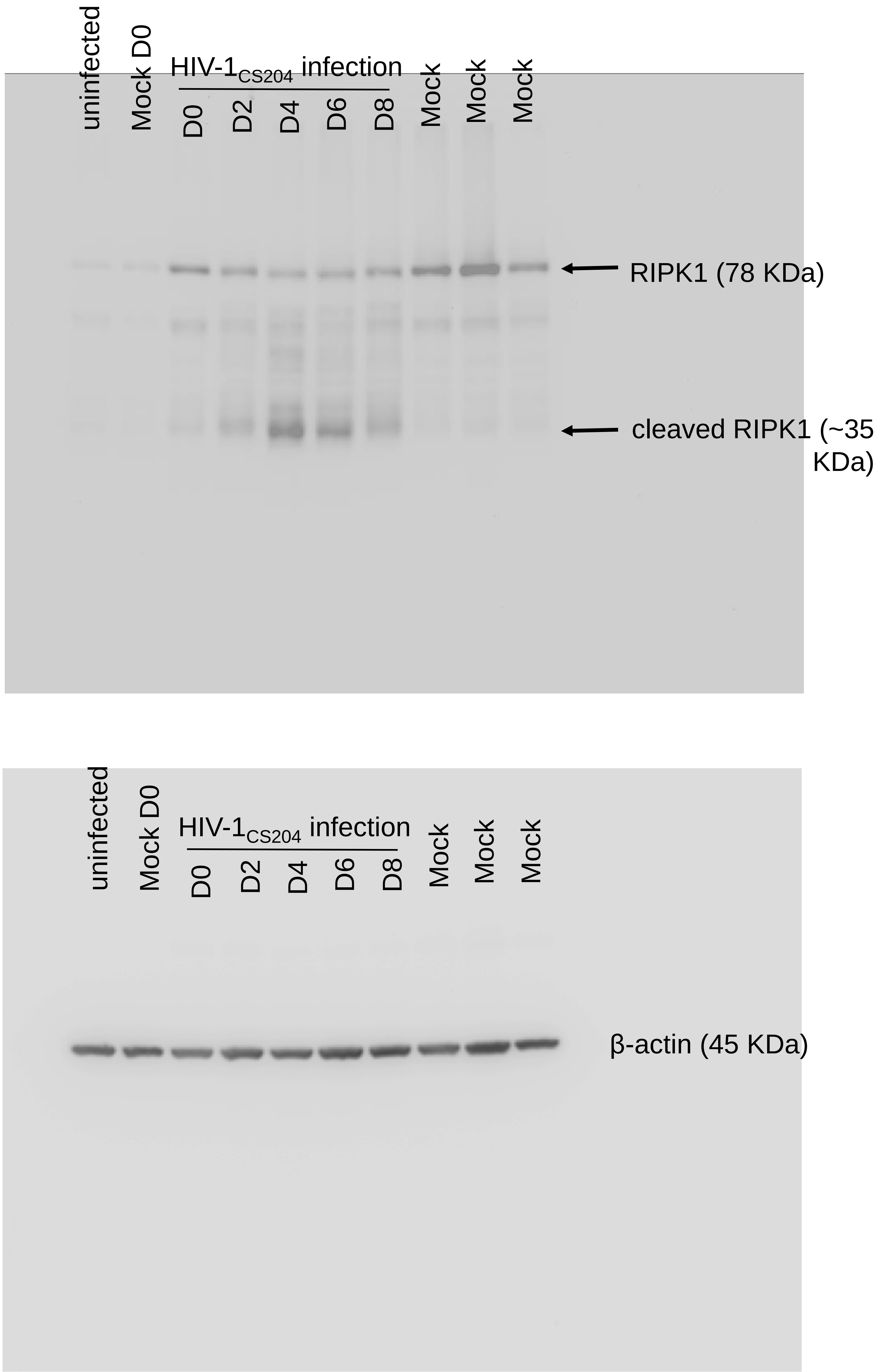

Fig 7B

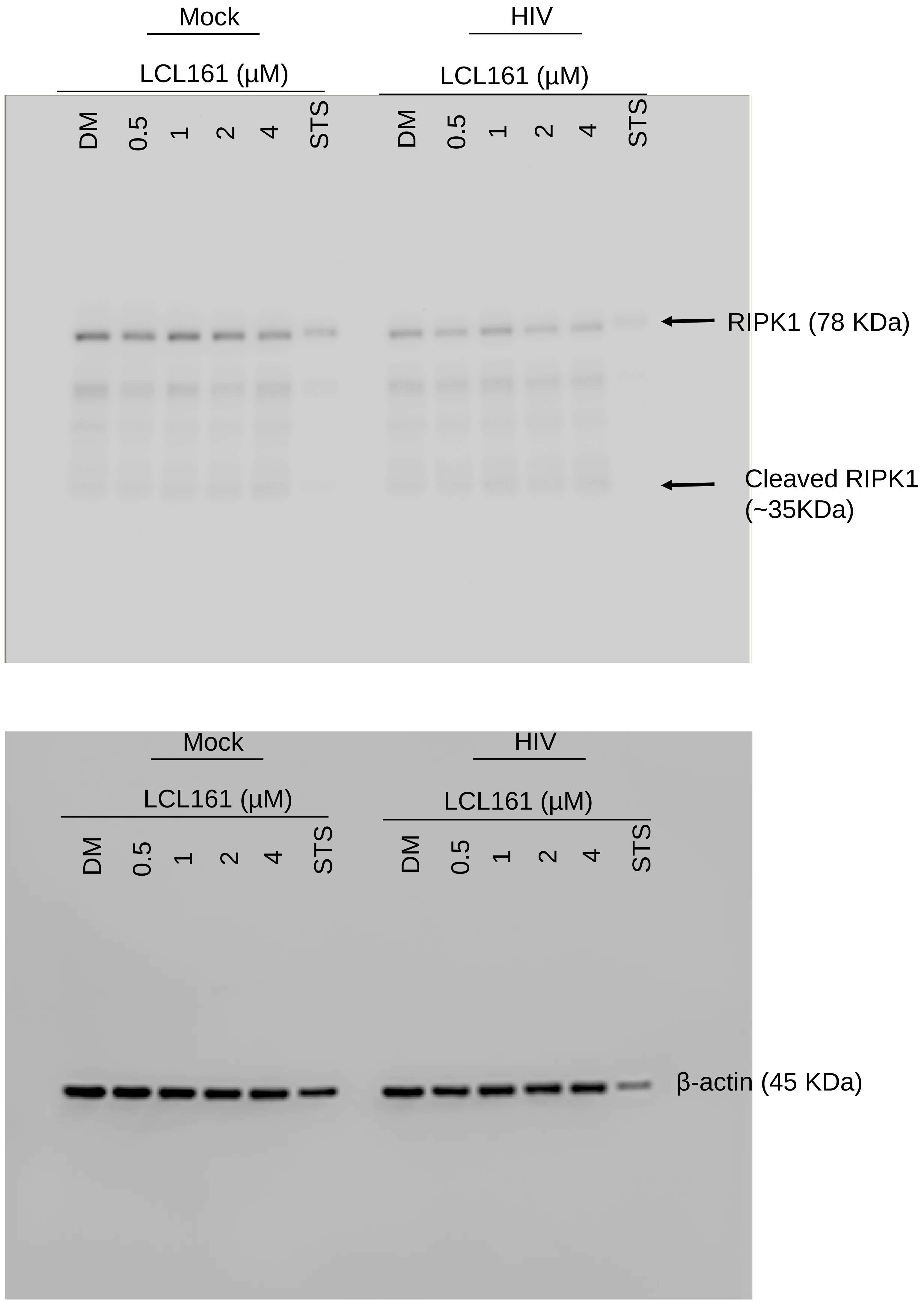

Suppl. Fig 8. Full blots for Fig7A and 7B for RIPK1 and beta-actin.

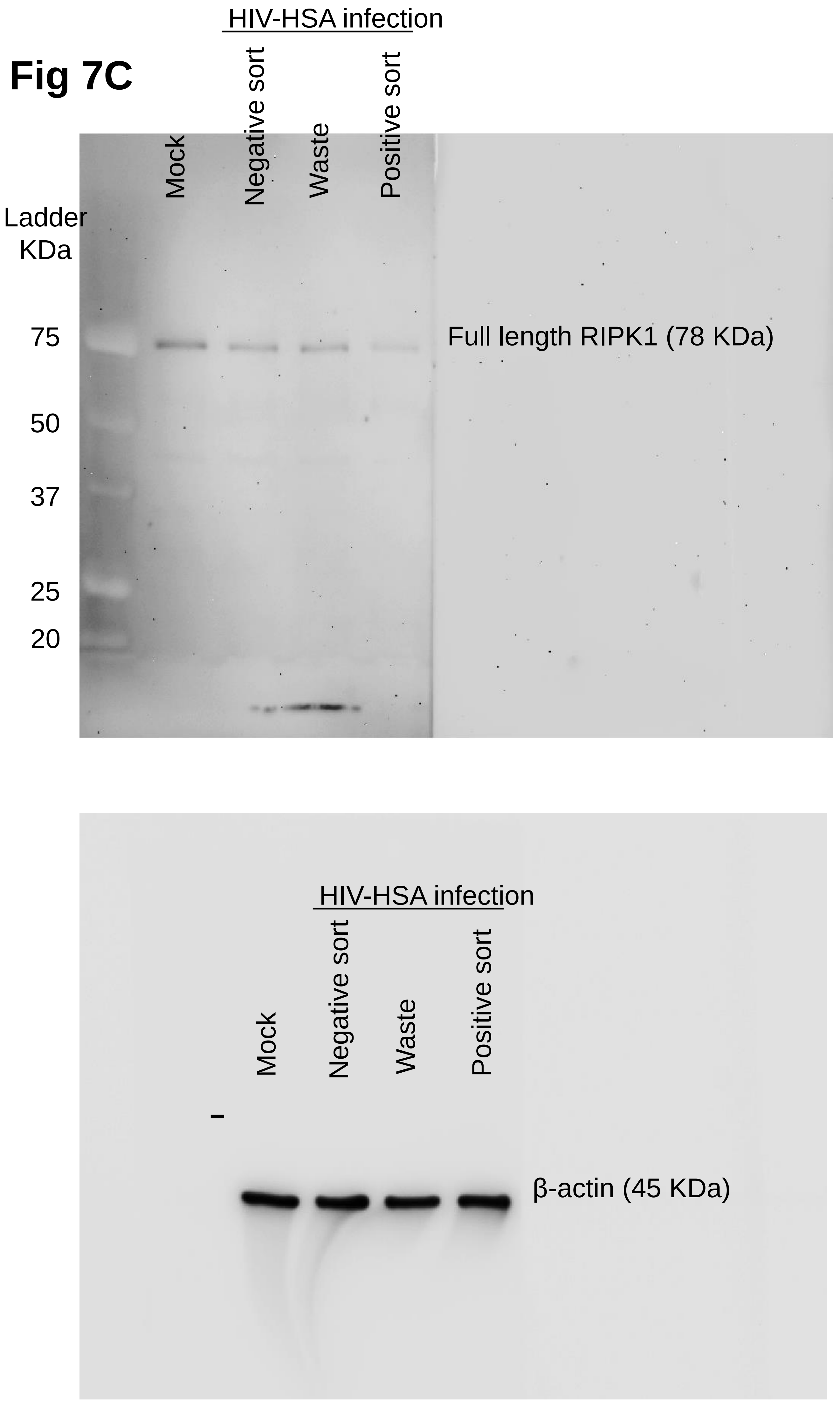

Suppl. Fig 9. Full blots for Fig7C for RIPK1 and beta-actin.

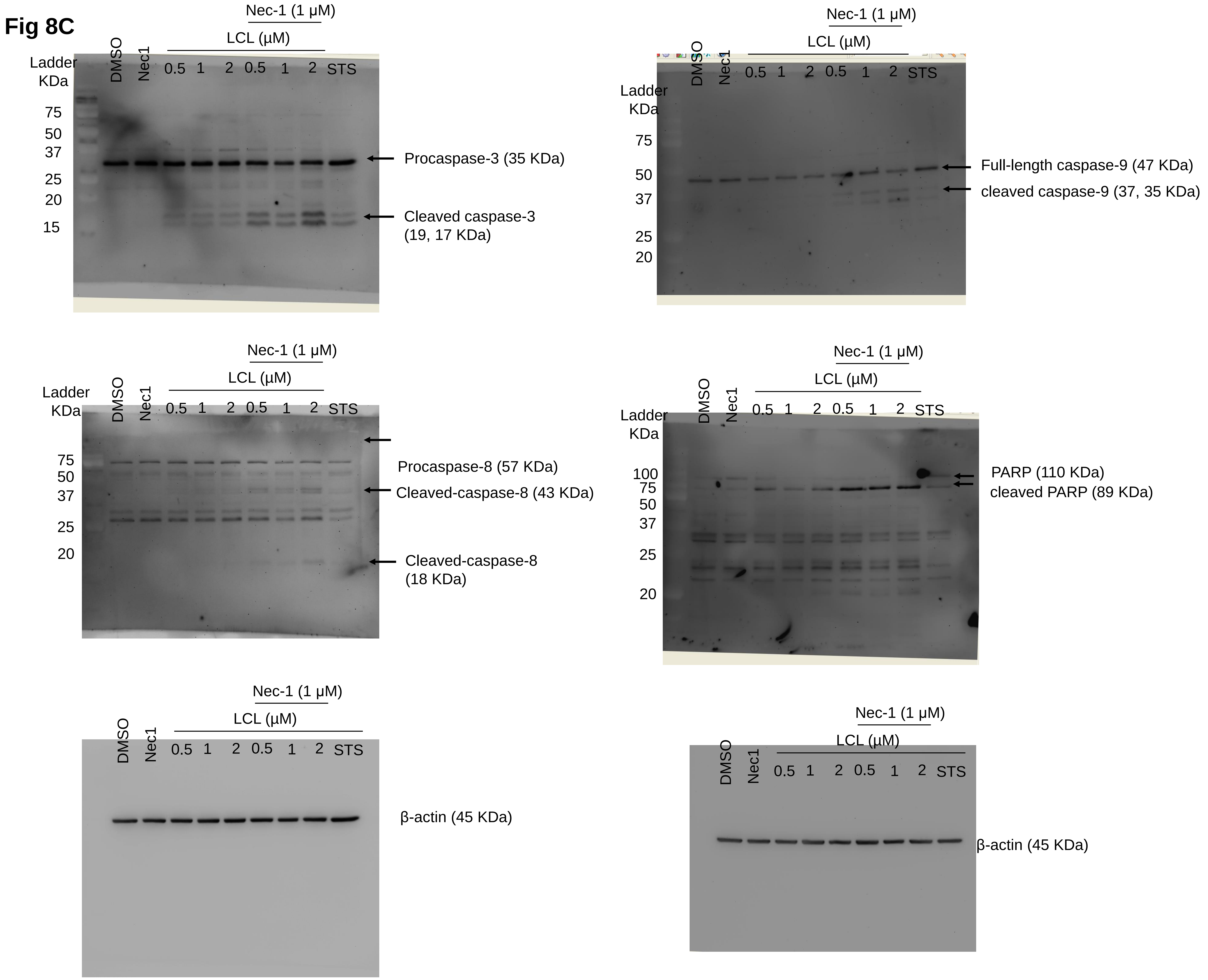

Suppl. Fig 10. Full blots for Fig8C caspase-3,-8,-9 and PARP and the corresponding blots for beta-actin.

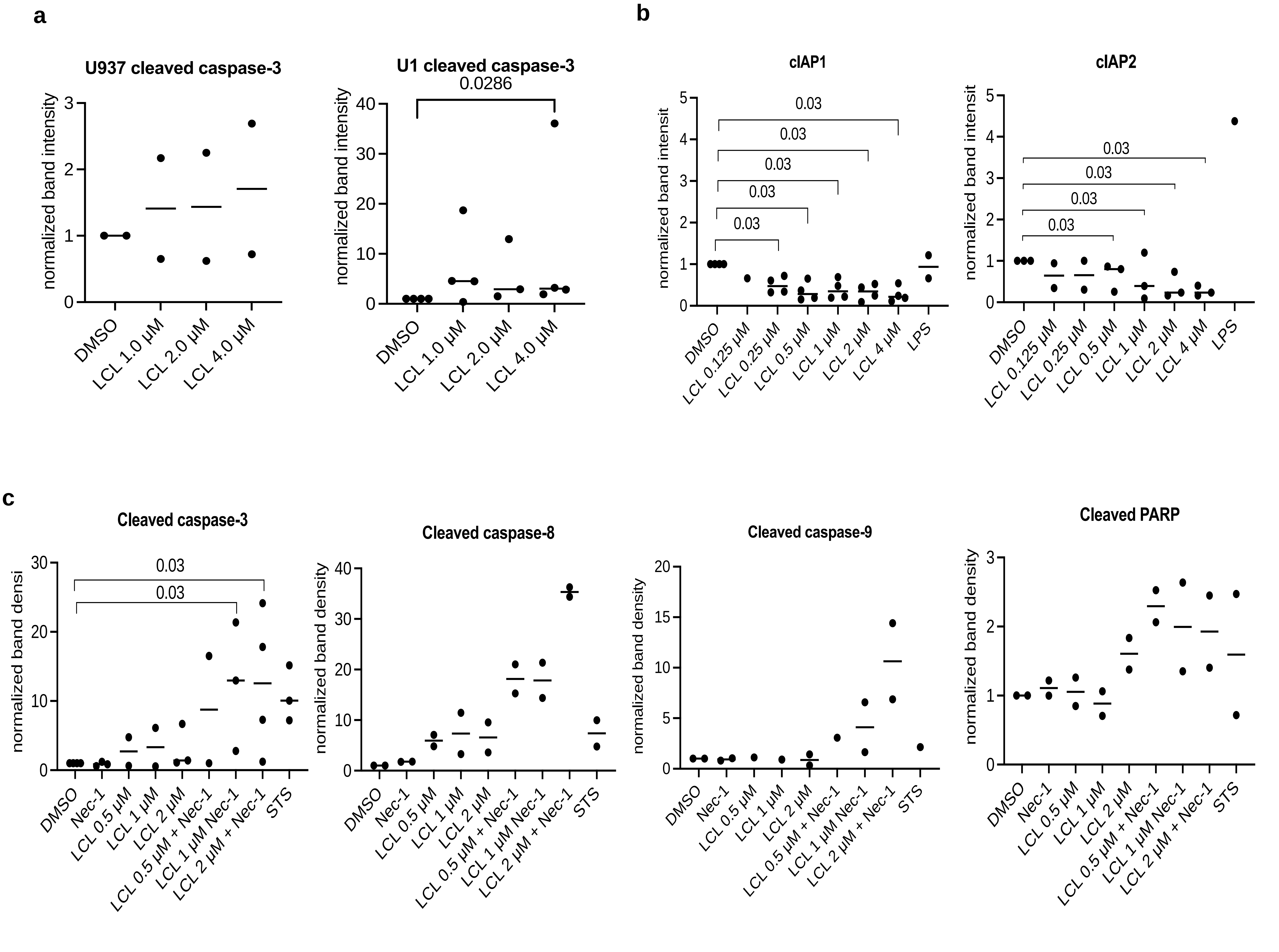

Suppl. Fig 11. Normalized band densities of western immunoblot experiments for Fig 1C, 2A, and 8C. (A) In figure 1C, LCL treatment increased the cleavage of executioner caspase-3 (17/19KDa) in U1 cells (technical replicate = 4). (B) In Fig 2A, increasing dose of LCL161 resulted in degradations of cIAP1 and cIAP1 in healthy MDMs (n=3). (C) LCL161 and necrostatin-1 treatment increased the cleavage of executioner caspase-3 (17/19 KDa) (n=3) and showed and increased trend in active cleaved caspase-8 (43 KDa) (n=2) and active cleaved caspase-9 (n=2) (35/37 KDa), as well as cleaved PARP (poly-ADP-ribose polymerase) (n=2) which is a marker of cell death. Two-tailed Mann-Whitney U test was used to calculate p values.

Supp Fig1b Donor 1

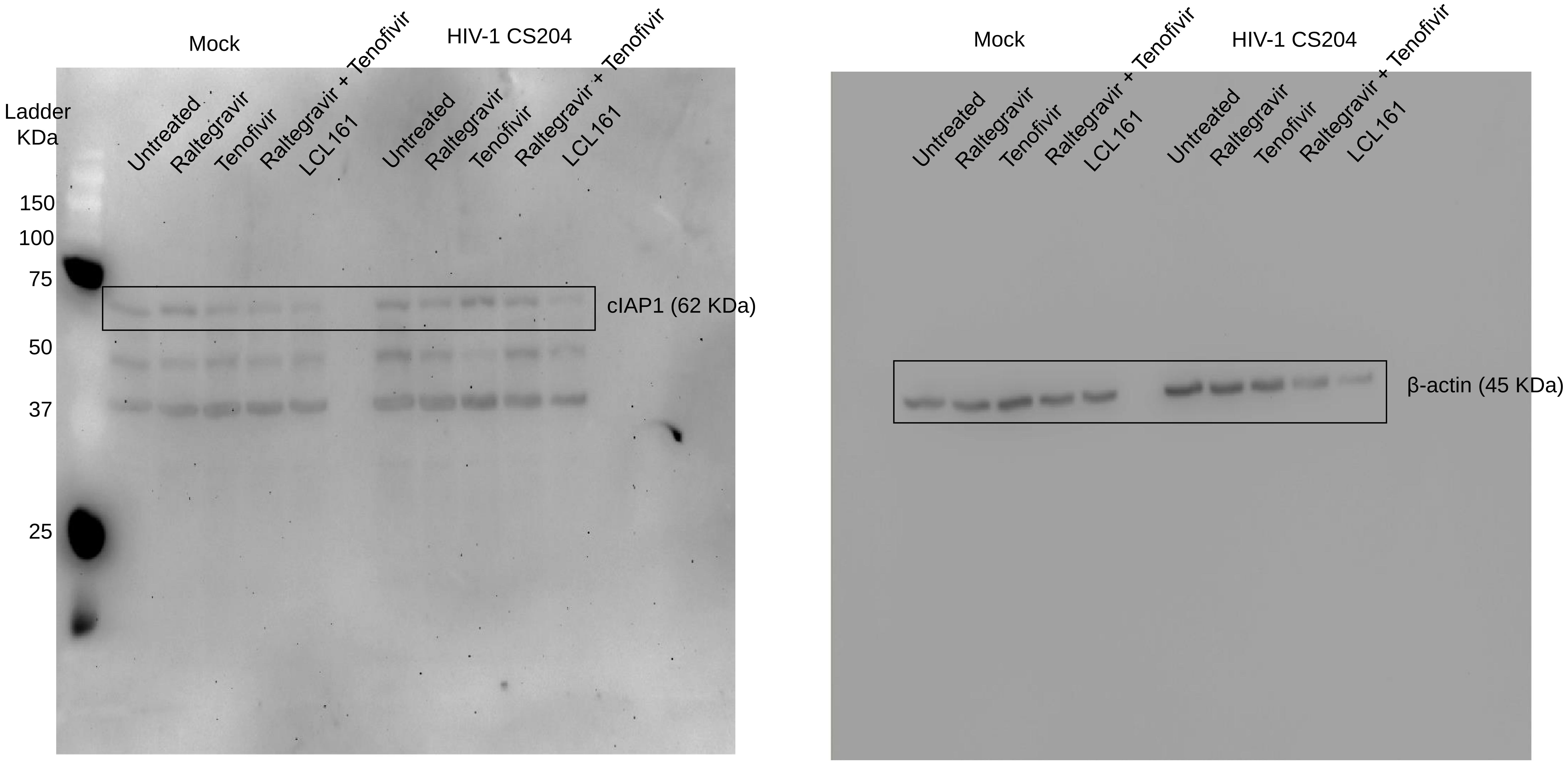

Supp Fig 12a. Uncropped blots for supplementary figure 1b donor 1 for cIAP1 and corresponding β-actin control (left panel, mock and HIV, upper 4 bands).

Supp Fig1b Donor 2

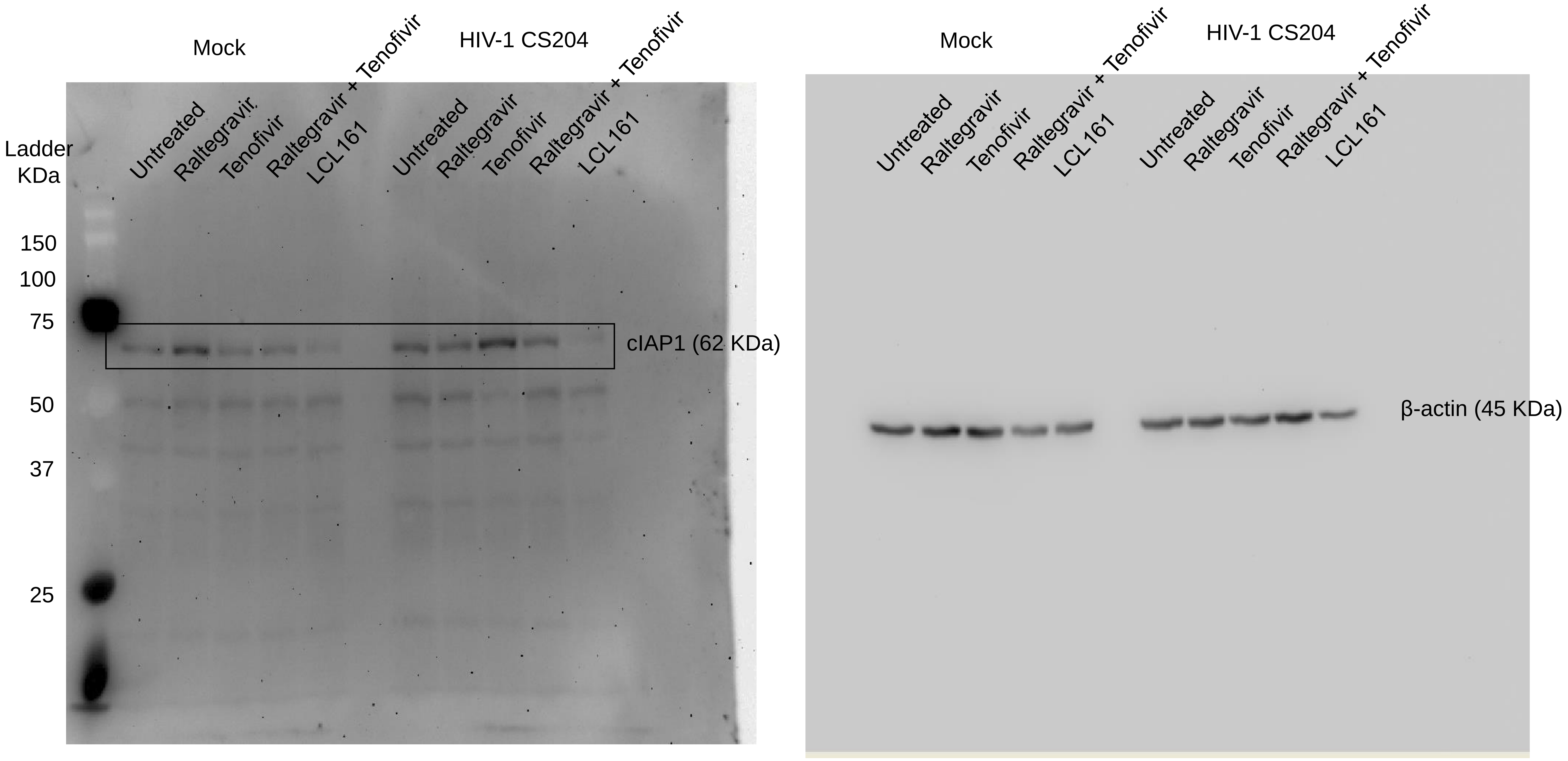

Supp Fig 12b. Uncropped blots for supplementary figure 1b donor 2 for cIAP1 and corresponding β-actin control (left panel, mock and HIV, middle 4 bands).

Supp Fig1b Donor 3

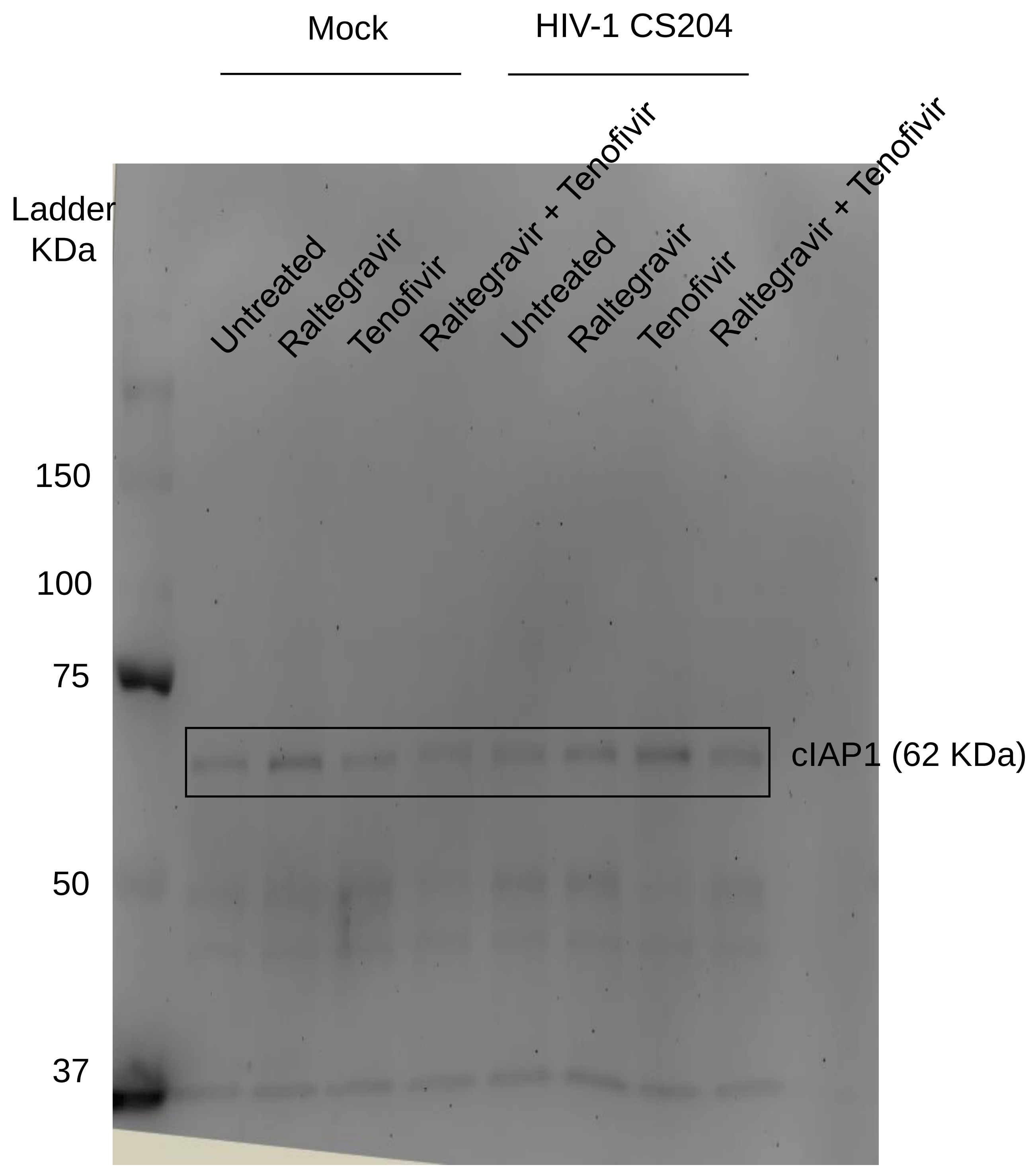

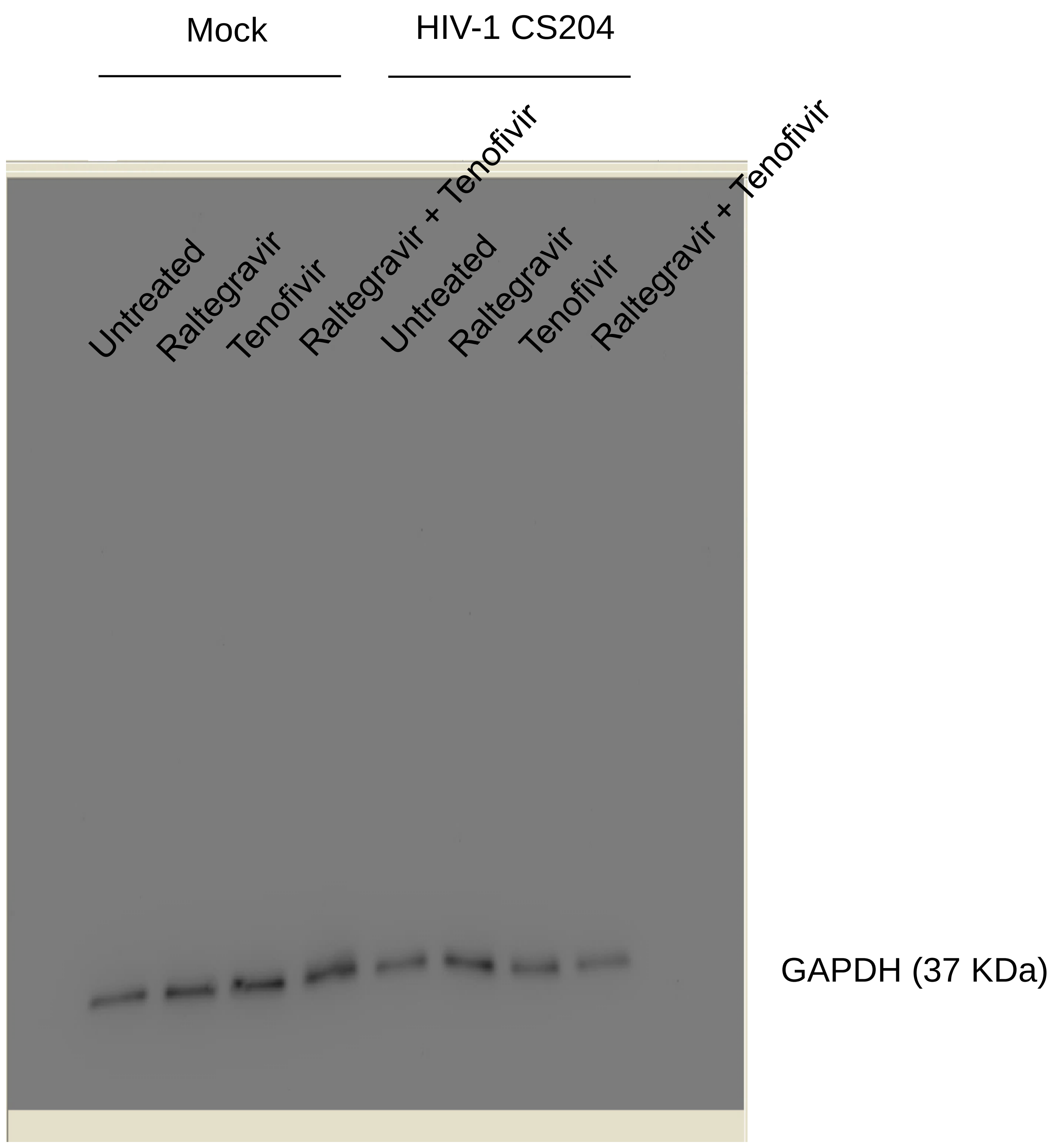

Supp Fig 12c. Uncropped blots for supplementary figure 1b donor 3 for cIAP1 and corresponding GAPDH control (left panel, mock and HIV, lower 4 bands).

Supp Fig1b Donor 1

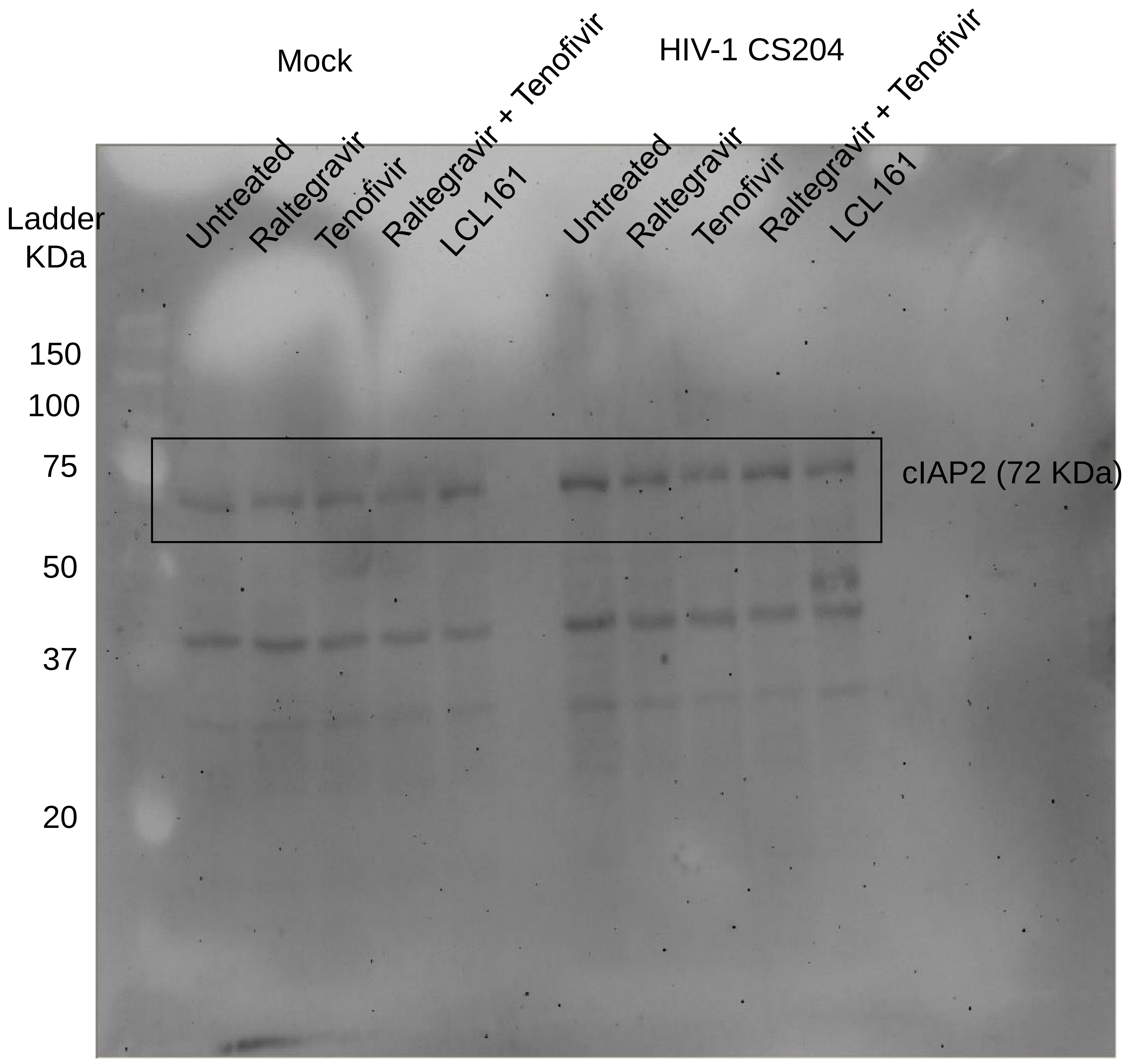

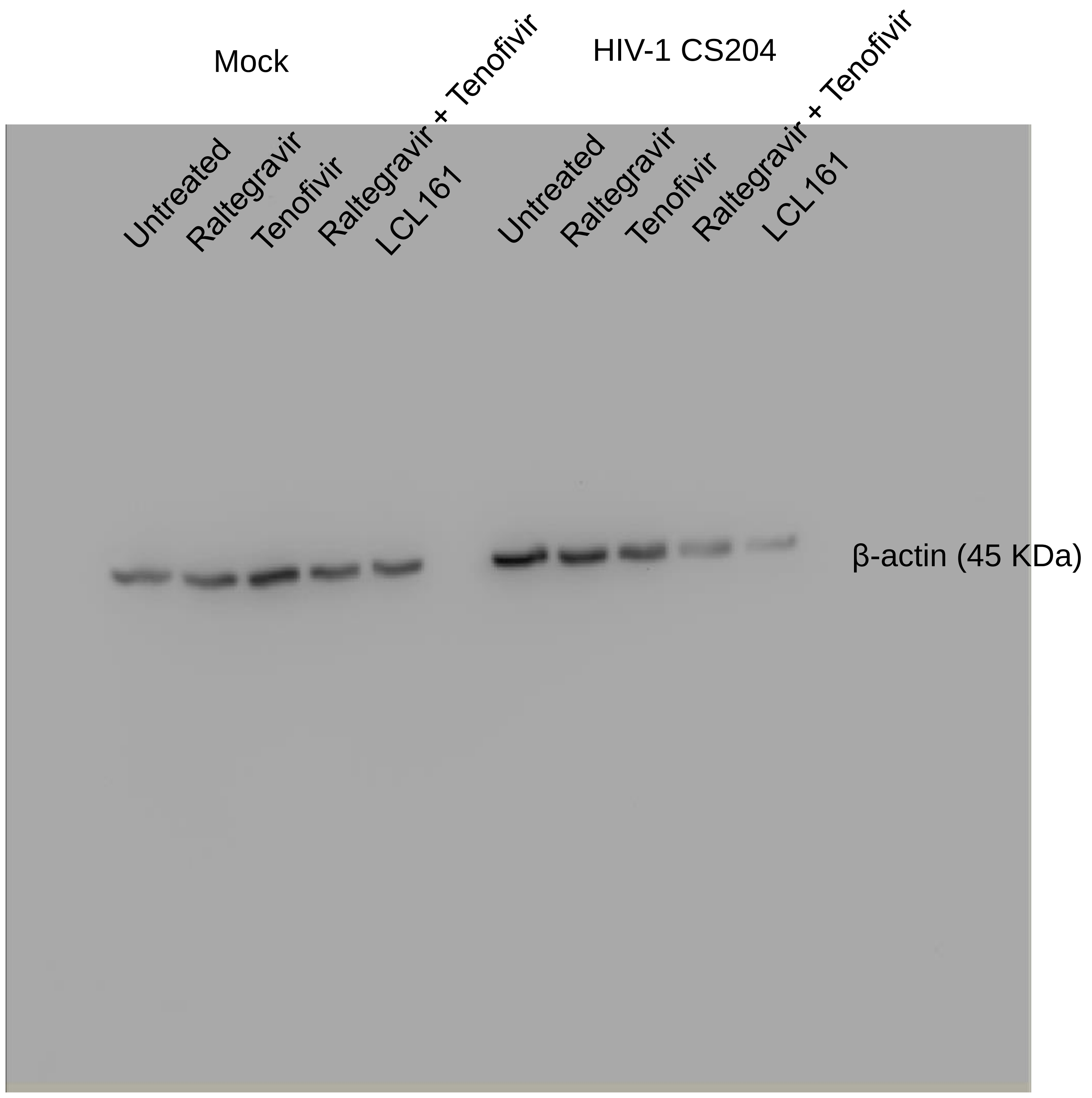

Supp Fig 12d. Uncropped blots for supplementary figure 1b donor 1 for cIAP2 and corresponding β-actin control (right panel, mock and HIV, upper 4 bands).

Supp Fig1b Donor 2

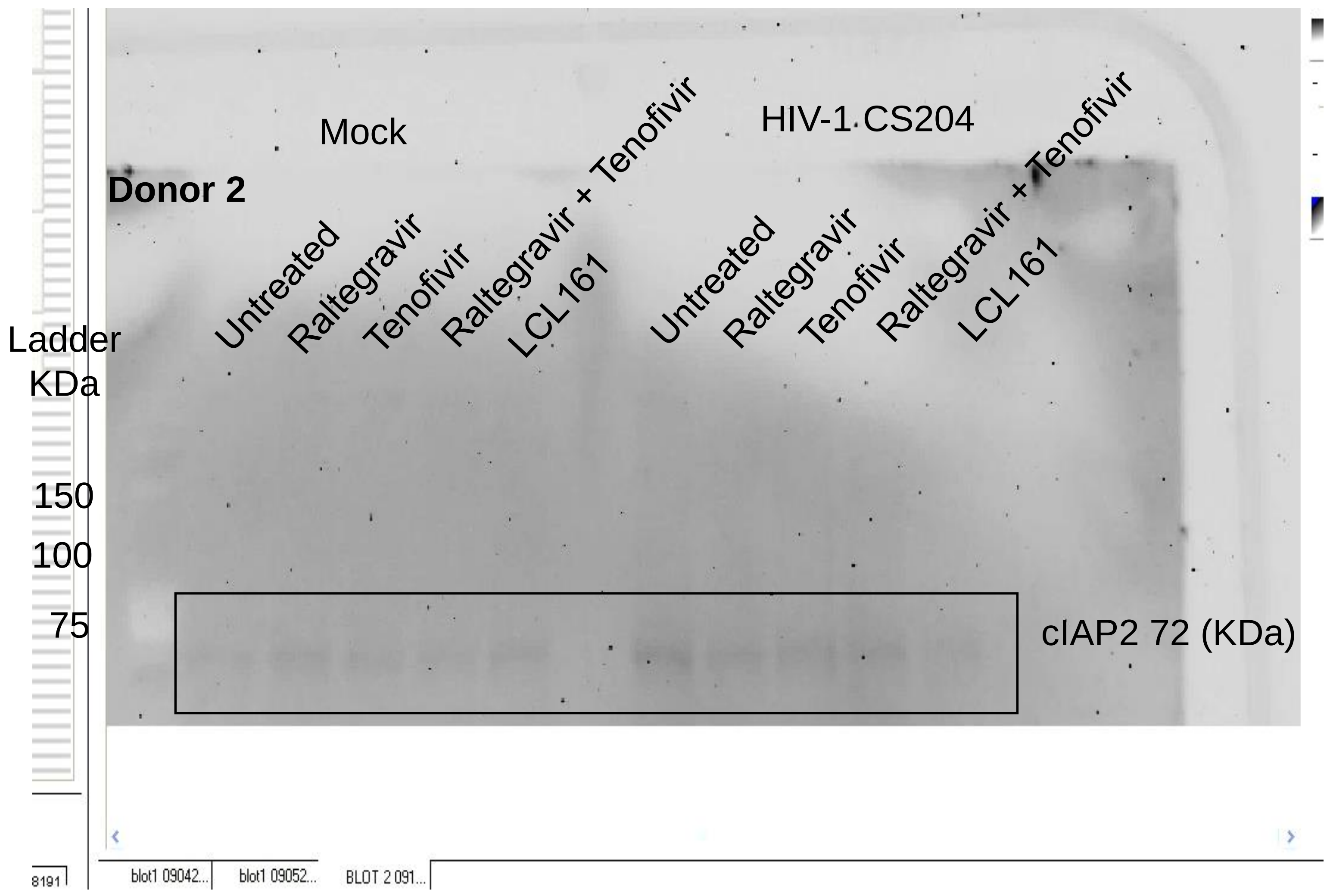

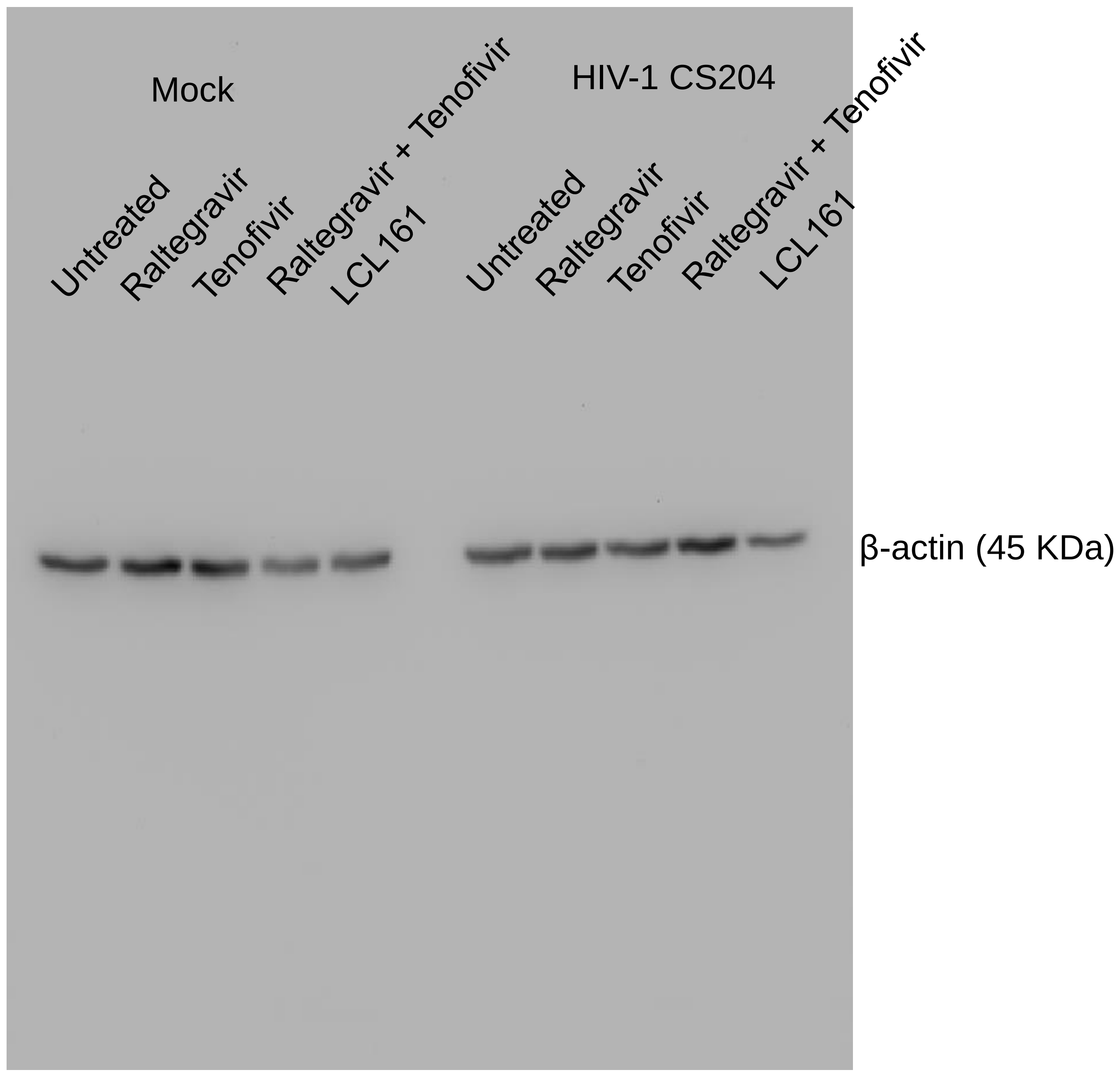

Supp Fig 12e. Uncropped blots for supplementary figure 1b donor 2 for cIAP2 and corresponding β-actin control (right panel, mock and HIV, middle 4 bands).

Supp Fig1b Donor 3

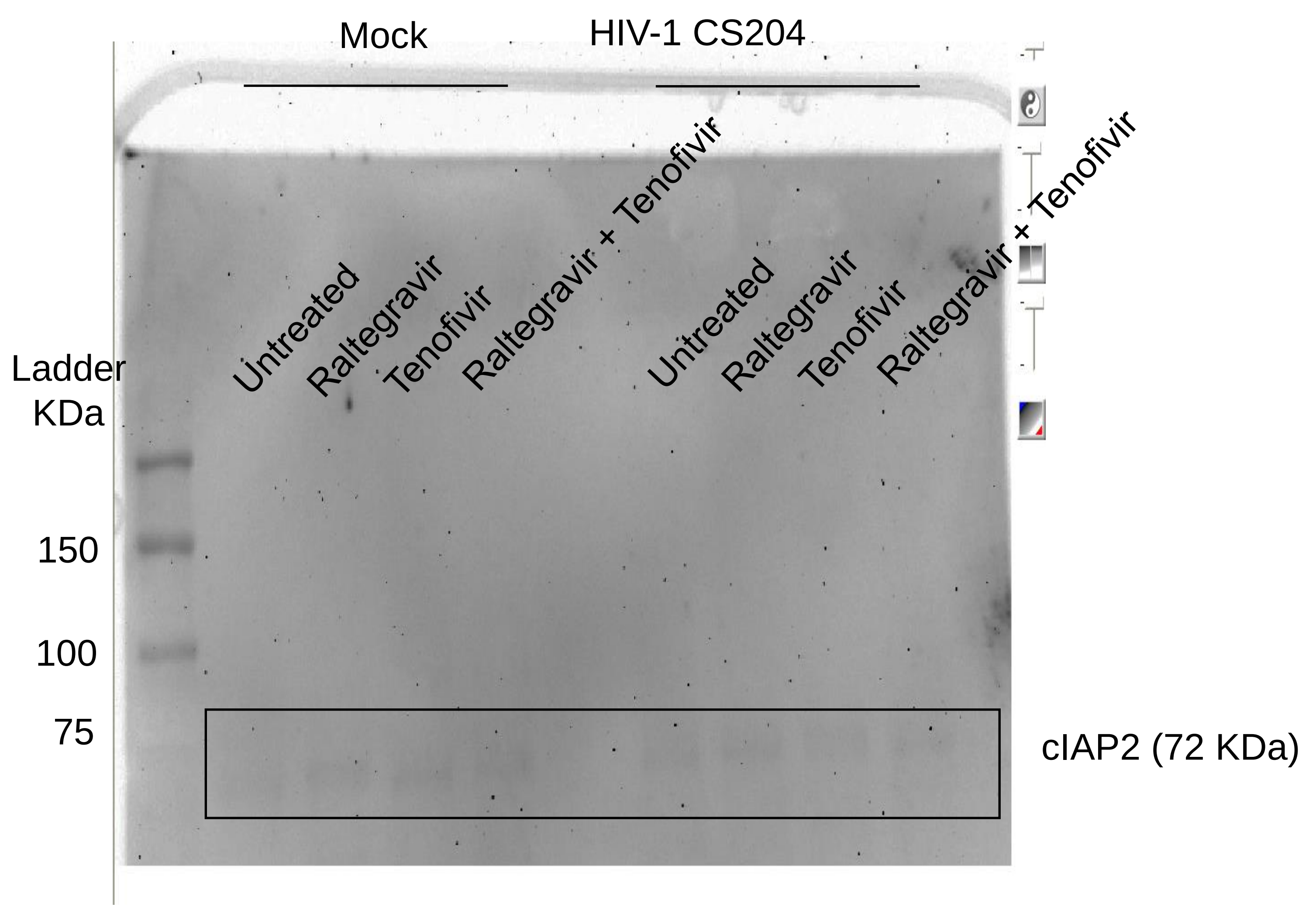

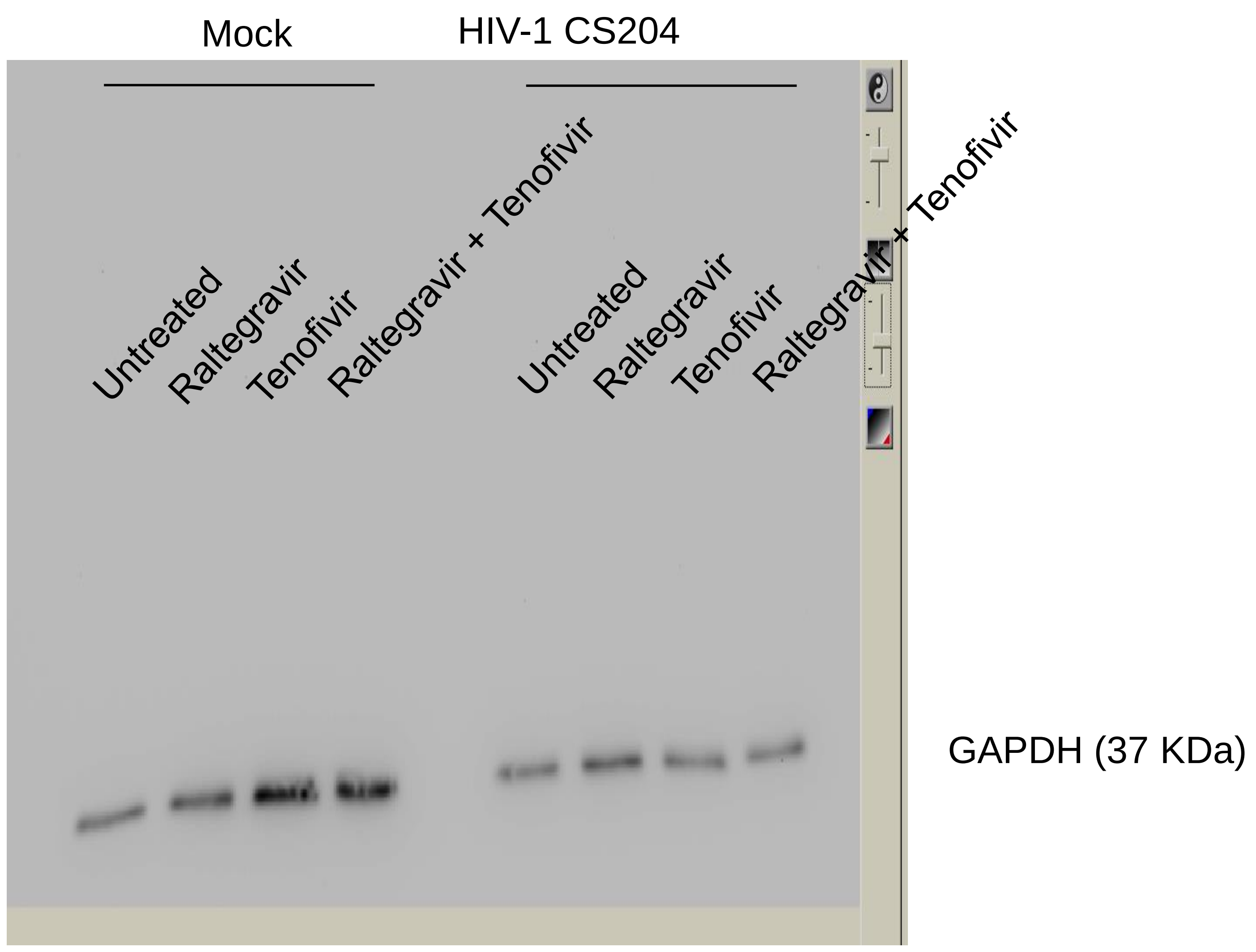

Supp Fig 12f. Uncropped blots for supplementary figure 1b donor 3 for cIAP2 and corresponding GAPDH control (right panel, mock and HIV, lower 4 bands).
